# Peripheral Blood Mononuclear Cells Profiling Revealed Biomarkers That Predict PD‐1 Inhibitor‐Induced Immune‐Related Adverse Events

**DOI:** 10.1002/mco2.70721

**Published:** 2026-04-05

**Authors:** Jun Wang, Hong Xie, Yuanyuan Gong, Songlin Liu, Yaping Guan, Yuekai Zhang, Qi Xie, Jingyi Wang, Ye Li, Xueqin Zeng, Xi Chen, Chen Wang

**Affiliations:** ^1^ Department of Oncology The First Affiliated Hospital of Shandong First Medical University & Shandong Provincial Qianfoshan Hospital Jinan China; ^2^ Shandong Lung Cancer Institute The First Affiliated Hospital of Shandong First Medical University & Shandong Provincial Qianfoshan Hospital Jinan China; ^3^ Lung Cancer Center The First Affiliated Hospital of Shandong First Medical University & Shandong Provincial Qianfoshan Hospital Jinan China; ^4^ Shanghai Key Laboratory of Molecular Imaging Pudong Gongli Hospital Shanghai University of Medicine and Health Sciences Shanghai China; ^5^ Shanghai Institute for Advanced Immunochemical Studies ShanghaiTech University Shanghai China; ^6^ The Second Department of Thoracic Oncology The Affiliated Cancer Hospital of Xiangya School of Medicine, Central South University/Hunan Cancer Hospital Changsha China; ^7^ Department of Oncology Senior Department of oncology The First Medical Center of Chinese PLA General Hospital Beijing China; ^8^ Pathology Department Renji Hospital of Shanghai Jiaotong University Shanghai China; ^9^ GMU‐GIBH Joint School of Life Sciences The Guangdong‐Hong Kong‐Macao Joint Laboratory for Cell Fate Regulation and Diseases Guangzhou Medical University Guangzhou China

**Keywords:** biomarker, C–C motif chemokine receptor 6, C–X–C motif chemokine receptor 3, immune checkpoint inhibitor, immune related adverse effect, programmed cell death 1

## Abstract

Immune checkpoint inhibitors (ICIs) universally enhance antitumor immunity but endanger a subgroup of patients by triggering immune‐related adverse events (irAEs). We profiled the expressions of 41 proteins on peripheral blood mononuclear cells (PBMCs) prior the initiation of immunotherapy. CXCR3 and CCR6 expressions were significantly decreased in PBMC subpopulations from patient with irAEs but not from those who responded to PD‐1 inhibitors. The expression of CCR6 in a NK cell subpopulation serves exclusively as a biomarker to differentiate patients who developed irAEs. Interestingly, circulating ligands of CXCR3, including CXCL9, CXCL10, and CXCL11, were significantly increased in patients who later developed irAEs after PD‐1 inhibitor treatment. The decreases of CXCR3 in three T cell subpopulations and decreases of CCR6 in a NK cell subpopulation were further validated in two independent external cohorts. Moreover, multiple proteins in PBMCs, distinct from the irAE‐predicting biomarkers, exhibited differential expression levels corresponding to the differential responses to the PD‐1 inhibitors. Via multiple independent cohorts, our study revealed crucial roles of CXCR3 and CCR6 in PD‐1‐induced irAEs, provided potential circulating biomarkers associated with toxicity and responses of PD‐1 inhibitors and further sculptured the landscape of immune cell heterogeneity via focusing on PBMC subpopulations.

## Graphical abstract

Prior to treatments, the decreased expression levels of CXCR3 in T cell subpopulations and decreased expression level of CCR6 in NK cell subpopulation sever as predictors of PD‐1 immunotherapy induced immune‐related adverse effects. Increased CXCL9, CXCL10, and CXCL11, three circulating ligands of CXCR3, are related to PD‐1 immunotherapy‐induced immune‐related adverse effects. Meanwhile, increased CD66b and Tim‐3 in monocyte subpopulations and the elevated percentage of CD27^high^CXCR3^high^ B cells predict favorable PD‐1 immunotherapy outcomes.

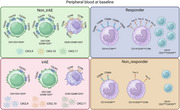

## 1 Introduction

Treatment with immune checkpoint inhibitors (ICIs), including programmed cell death protein‐1 (PD‐1)/programmed cell death ligand‐1 (PD‐L1) antibodies, has been developed as a milestone of systematic therapy for various malignancies at the early, local advanced, and metastatic stages [1]. PD‐1 was initially identified for its mRNA upregulation after induction of classical programmed cell death [2]. Before the tremendous anticancer potential of PD‐1 was raised [3, 4], researchers focused on the immunoinhibitory role of PD‐1 in lymphocytes. Studies showed that PD‐1‐deficient mice developed glomerulonephritis and arthritis in 14 months [5] and started to die as early as 5 weeks of age from heart failure resulted from autoimmune dilated cardiomyopathy [6]. Similar to PD‐1 deficiency, immunotherapy with PD‐1/PD‐L1 inhibitors also lead to autoimmune‐like or inflammatory conditions, namely, immune‐related adverse events (irAEs). The majority of irAEs are mild to moderate but in a minority of patients, irAEs are severe and even life threatening. In an analysis based on VigiBase, 31,492 irAEs were reported in 91,888 patients with anti‐PD‐1 monotherapy, while approximately 11.8% fatality was caused by irAEs [7]. The alerting datasets urge researchers to identify biomarkers that differentiate the patients with high risks of irAEs and to refine the regimen accordingly.

The heterogeneity of immune cell constitutions and status [8, 9, 10] as well as germline variants and microbiota diversity [11, 12, 13] contributed to irAE occurrence and numerous biomarkers aiming at predicting irAEs have been proposed [14, 15]. Single‐cell RNA sequencing (scRNA‐seq) and cytometry by time of flight (CyTOF) enrich the toolbox to study the heterogeneity of immune cells that could be responsible for the occurrences of irAEs and responses to ICI treatment [16, 17]. CyTOF has long been authenticated as a powerful tool in resolving the heterogeneity of immune cell landscapes during pharmacologic intervention [18], cancer immunotherapy [19], as well as adaptive immune responses [20]. Since CyTOF directly detects the protein levels in PBMCs, it provides immediate alterations of immune cells, which provide expedient and ample information that facilitates the development, monitoring as well as evaluation of immune‐checkpoint cancer therapy [21, 22]. Recently, it has been shown that CyTOF‐based single circulating immune cell analysis contributed in the identification of crucial biomarkers associated with occurrences of irAEs resulted from ICI regimens [16, 23].

C–X–C motif chemokine receptor 3 (CXCR3) serves as the receptor of CXC chemokine IP10 (C–X–C motif chemokine ligand 10; CXCL10) and MIG (C–X–C motif chemokine ligand 9; CXCL9) [24, 25], while CC chemokine receptor 6 (CCR6) serves as the receptor of CC chemokine liver and activation‐regulated chemokine [26, 27]. Both receptors are G protein‐coupled receptors and play fundamental roles in immunity. CXCR3‐deficient mice showed profound resistance to development of acute allograft rejection [28] and CXCR3‐deficient T cells failed in locating, engaging, and killing virus‐infected cells [29]. Importantly, a recent report showed that CXCR3‐deficient mice exhibited poor responses to anti‐PD‐1 treatment suggesting that the CXCR3 chemokine system is associated with the sensitivity to PD‐1 blockade [30]. Similarly, CCR6 was found responsible for recruiting alloreactive CD4^+^ T cells to target tissues in acute graft‐versus‐host disease [31] and CCR6‐deficient mice developed less clinical signs of arthritis in the collagen‐induced arthritis model [32]. In a mice model of pulmonary melanoma, CCR6 deficiency abrogated the Th9 cell‐mediated antitumor response [33].

To decipher the heterogeneity of immune cell landscapes related to occurrences of irAE post‐PD‐1 inhibitor treatment, we employed a 32 pan‐cancer patient cohort who received PD‐1 inhibitor therapy and analyzed 21 PBMCs collected before treatment with CyTOF via 41 protein markers. To elucidate the differences of immune cells between patient who developed irAE (irAE group) after treatment from those without irAE (non_irAE group), we analyzed the differentially expressed protein markers in overall PBMCs as well as in immune cell subpopulations and found that CXCR3 and CCR6 expressions were significantly decreased in multiple immune cell populations. We also identified a subpopulation of HLA‐DR^+^CD11c^+^CXCR3^+^CD3^−^CD19^−^CD14^−^CD56^−^ dendritic cells, which is absent in the irAE group. We further examined the circulating ligands of CXCR3 and CCR6 and augmented four more tissue samples to detect the presence of CXCR3 expressing T cells. Additionally, the cohort was further divided into partial response (PR; the group of patients that are responsive to PD‐1 inhibitors) and stable disease/progressive disease group (SD/PD; the group that are nonresponsive to PD‐1 inhibitors) and differentially expressed protein markers as well as immune cell subpopulations were identified.

## 2 Results

### 2.1 Identification of Differentially Expressed Protein Markers in PBMC Between irAE and Non_irAE Group

**FIGURE 1:**
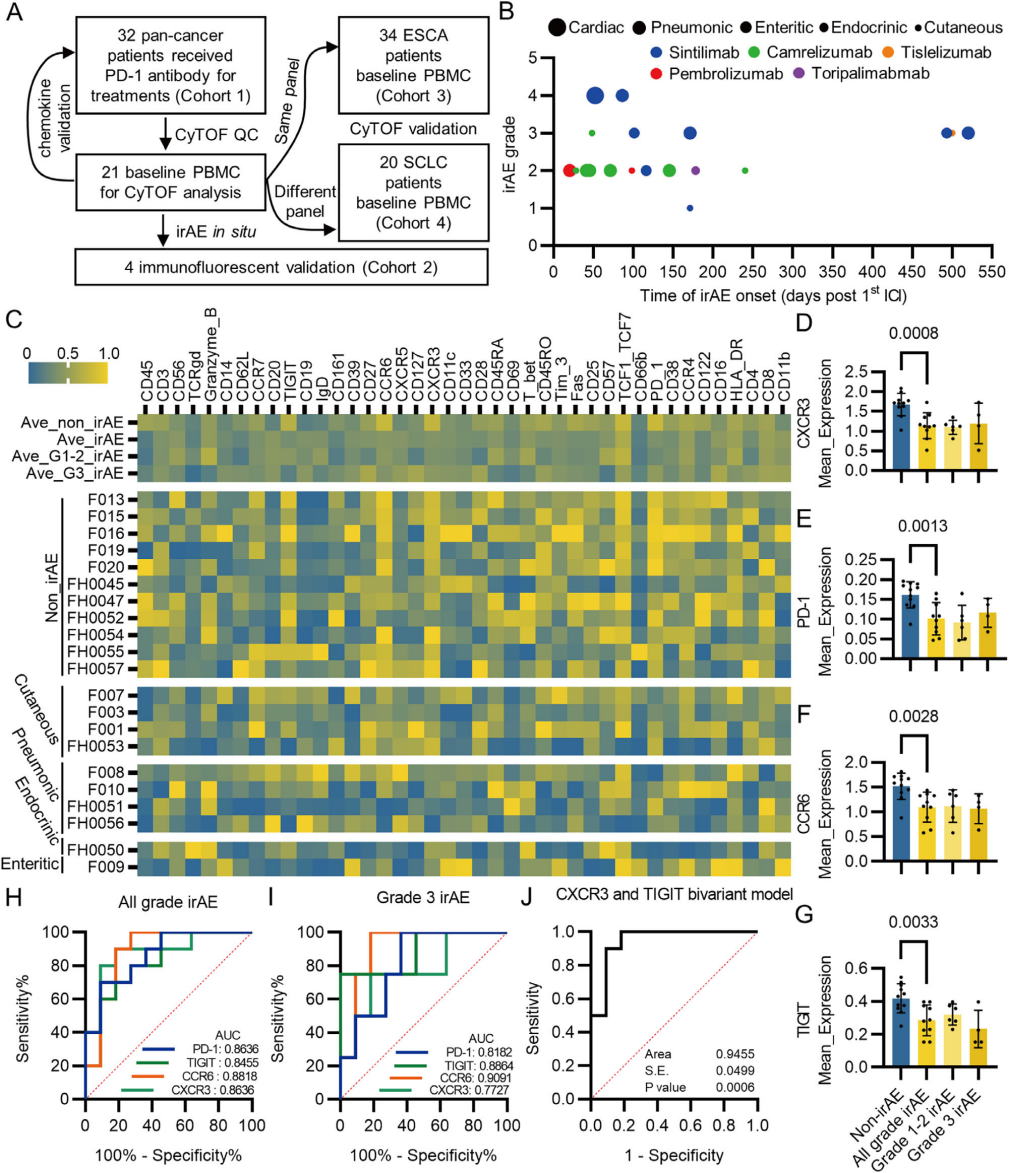
Identification of differentially expressed protein markers between irAE and non_irAE group via CyTOF analysis. (A) Cohort and experimental design. (B) Characteristics of irAE occurrences, types, and grades. (C) Heatmap of protein markers of 21 patients subjected to CyTOF grouped by irAE status. (D) CXCR3 expression levels in non_irAE group, irAE group, Grade 1–1 irAE group, and Grade 3 irAE group. (E) PD‐1 expression levels in non_irAE group, irAE group, Grade 1–1 irAE group, and Grade 3 irAE group. (F) CCR6 expression levels in non_irAE group, irAE group, Grade 1–1 irAE group, and Grade 3 irAE group. (G) TIGIT expression levels in non_irAE group, irAE group, Grade 1–1 irAE group, and Grade 3 irAE group. (H) ROC curves and AUCs of PD‐1 (AUC: 0.8636, 95% CI: 0.7089–1.000, *p* value: 0.0049), TIGIT (AUC: 0.8455, 95% CI: 0.6806–1.000, *p* value: 0.0075), CCR6 (AUC: 0.8818, 95% CI: 0.7264–1.000, *p* value: 0.0031), and CXCR3 (AUC: 0.8636, 95% CI: 0.6914–1.000, *p* value: 0.0049) in predicting occurrences of all grades irAEs. (I) ROC curves and AUCs of PD‐1 (AUC: 0.8182, 95% CI: 0.5965–1.000, *p* value: 0.0676), TIGIT (AUC: 0.8864, 95% CI: 0.6703–1.000, *p* value: 0.0265), CCR6 (AUC: 0.9091, 95% CI: 0.7557–1.000, *p* value: 0.0188), and CXCR3 (AUC: 0.7727, 95% CI: 0.4883–1.000, *p* value: 0.1172) in predicting occurrences of Grade 3 irAEs. (J) ROC curve and AUC of CXCR3 and TIGIT bivariant model (AUC: 0.9455, 95% CI: 0.8477–1.000, *p* value: 0.0006) in predicting occurrence of all grade irAEs.

To identify specific PBMC populations and proteins that distinguish patients who are at the risk for developing irAEs, we employed a cohort (Cohort 1, discovery cohort) consisting of 32 pan‐cancer patients who received PD‐1 inhibitor treatment (Figure 1A and Tables 1 and S1). Of 15 patients with irAEs, one patient developed Grade 1 immune‐related rash, six patients developed Grade 2 irAEs, and six patients developed Grade 3 irAEs. One patient developed Grade 4 myocarditis and another developed Grade 4 pneumonia (Figure 1B). Most irAEs arose within half a year following the treatment of immunotherapy. 40% (six out of 15) of irAEs were early‐onset irAEs (at 0–90 days after ICI initiation) and 60% were late‐onset irAEs (at more than 90 days after ICI initiation). Among the early‐onset irAEs, four were pneumonia, accounting for 60% (four out of seven) of overall pneumonia, while only one was cutaneous irAE accounting for 25% (one out of four) of overall cutaneous irAEs. Moreover, endocrine (one) and enteritic (two) happened after 3 months of ICI treatments, while one myocarditis happened 52 days post 1st ICI treatment. 50% (three out of six) of early‐onset irAEs were Grade 3 or higher, while 55.5% (five out of 10) of late‐onset irAEs were Grade 3 (Figure 1B). A total of three patients developed multiple irAEs (Table S1). After quality control of the thawed PBMCs, 21 samples were subjected to CyTOF analysis (Figure 1C and Table S2). At holistic level, CXCR3, PD‐1, CCR6, TIGIT, Fas, and CD25 expressions were significantly decreased in the irAE group compared with the non_irAE group (Figures 1D–G and S1A,B,D). The expression levels of CXCR3, PD‐1, CCR6, and TIGIT, but not Fas and CD25, showed superior potentials in predicating irAEs with all grades or Grade 3 (Figures 1H,I and S1C,E). A bivariant model of CXCR3 and TIGIT expression levels showed a prominent efficacy in predicting all grades irAEs with an area under the receiver operating characteristic curve (AUC) of 0.9455 (Figure 1J).

**TABLE 1:** Demographics and clinical characteristics.

	Non_irAE	irAE	PR	SD/PD
Number of patients	17	15	11	18
ECOG				
0	4	3	2	5
1	9	12	8	11
2	4	0	1	2
Diagnosis				
Large‐cell neuroendocrine carcinoma of the lung	1	0	0	1
Kidney renal clear cell carcinoma	1	3	2	2
Lung adenocarcinoma	3	4	4	3
Lung squamous carcinoma	1	3	2	2
Stomach adenocarcinoma	8	3	3	8
Cholangiocarcinoma	1	0	0	1
Melanoma	0	1	0	1
Colon adenocarcinoma	0	1	0	1
Small cell lung cancer	1	0	0	1
Squamous cell carcinoma of skin	1	0	0	1
PD‐1 inhibitor				
Sintilimab	13	7	5	7
Tislelizumab	1	1	1	1
Camrelizumab	3	5	1	4
Pembrolizumab	0	1	0	1
Toripalimab	0	1	0	1
irAE grade				
0	0	0	4	10
1	0	1	1	0
2	0	6	1	5
3	0	6	5	1
4	0	2	0	2
Response				
PR	4	7	11	0
SD/PD	13	8	0	18
Average_Age	63.7	59.0	61.2	61.5
Average_BMI	22.9	26.5	25.8	24.5

### 2.2 CXCR3 and CCR6 Were Decreased in PBMC Subpopulations from Patients Who Developed irAEs

**FIGURE 2:**
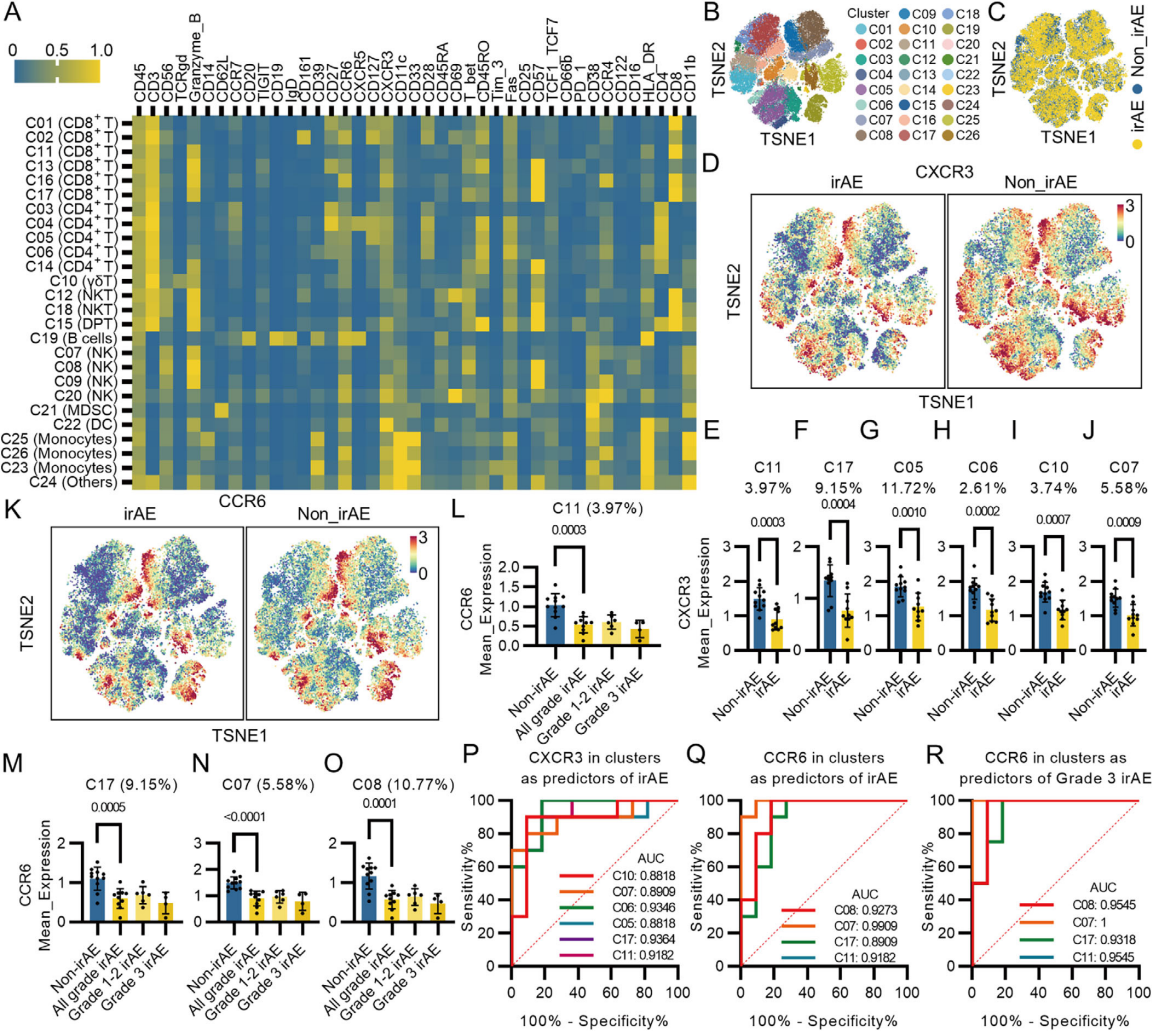
CXCR3 and CCR6 were decreased in PBMC subpopulations from patients developed irAEs. (A) Heatmap illustrating 26 clusters identified based on the expressions of protein markers of 11 non_irAE patients. (B) TSNE plot illustrating the 26 clusters. (C) TSNE plot illustrating the differential distributions of CyTOF events between irAE and non_irAE groups. (D) TSNE plots illustrating the differential expression levels and distributions of CXCR3 between irAE and non_irAE groups. (E) Expression levels of CXCR3 in Cluster 11 whose average percentage of total PBMS is 3.97% between irAE and non_irAE groups. (F) Expression levels of CXCR3 in Cluster 17 between irAE and non_irAE groups. (G) Expression levels of CXCR3 in Cluster 05 between irAE and non_irAE groups. (H) Expression levels of CXCR3 in Cluster 06 between irAE and non_irAE groups. (I) Expression levels of CXCR3 in Cluster 10 between irAE and non_irAE groups. (J) Expression levels of CXCR3 in Cluster 07 between irAE and non_irAE groups. (K) TSNE plots illustrating the differential expression levels and distributions of CCR6 between irAE and non_irAE groups. (L) Expression levels of CCR6 in Cluster 11 among non_irAE, all grades irAE, Grade 1–2 irAE, and Grade 3 groups. (M) Expression levels of CCR6 in Cluster 17 among non_irAE, all grades irAE, Grade 1–2 irAE, and Grade 3 groups. (N) Expression levels of CCR6 in Cluster 07 among non_irAE, all grades irAE, Grade 1–2 irAE and Grade 3 groups. (O) Expression levels of CCR6 in Cluster 08 among non_irAE, all grades irAE, Grade 1–2 irAE, and Grade 3 groups. (P) ROC curves and AUCs of CXCR3 in C10 (AUC: 0.8818, 95% CI: 0.7207–1.000, *p* value: 0.0031), CXCR3 in C07 (AUC: 0.8909, 95% CI: 0.7388–1.000, *p* value: 0.0025), CXCR3 in C06 (AUC: 0.9364, 95% CI: 0.8349–1.000, *p* value: 0.0007), CXCR3 in C05 (AUC: 0.8818, 95% CI: 0.7169–1.000, *p* value: 0.0031), CXCR3 in C17 (AUC: 0.9364, 95% CI: 0.8349–1.000, *p* value: 0.0007), and CXCR3 in C11 (AUC: 0.9182, 95% CI: 0.8037–1.000, *p* value: 0.0012), in predicting occurrences of all grades irAEs. (Q) ROC curves and AUCs of CCR6 in C08 (AUC: 0.9273, 95% CI: 0.8101–1.000, *p* value: 0.0009), CCR6 in C07 (AUC: 0.9909, 95% CI: 0.9620–1.000, *p* value: 0.0001), CCR6 in C17 (AUC: 0.8909, 95% CI: 0.7450–1.000, *p* value: 0.0025), and CCR6 in C11 (AUC: 0.9182, 95% CI: 0.7875–1.000, *p* value: 0.0012) in predicting occurrences of all grades irAEs. (R) ROC curves and AUCs of CCR6 in C08 (AUC: 0.9545, 95% CI: 0.8496–1.000, *p* value: 0.0090), CCR6 in C07 (AUC: 1.000, 95% CI: 1.000–1.000, *p* value: 0.0041), CCR6 in C17 (AUC: 0.9318, 95% CI: 0.8025–1.000, *p* value: 0.0131), and CCR6 in C11 (AUC: 0.9545, 95% CI: 0.8496–1.000, *p* value: 0.0090) in predicting occurrences of all Grade 3 irAEs.

PBMCs were further clustered into 26 subpopulations through dimensionality reduction and clustering based on the expression of the protein markers of patients without irAEs (Figures 2A,B and S2), which revealed differences in frequencies of clusters as shown by the *t*‐distributed stochastic neighbor embedding (TSNE) plot (Figures 2C and S3). Intriguingly, we found that CXCR3 and CCR6 (Figures 2D–O and S4A–K) as well as PD‐1, TIGIT, Fas, and CD25 (Figures S5 and S6) were differentially expressed in a series of PBMC clusters. CXCR3 expressions were significantly decreased in CD8^+^ T cells (C01, C11, C13, C17), CD4^+^ T cells (C05, C06, C14), γδT cell (C10), NK cells (C07, C08), and B cells (C19) (Figures 2E–J and S4A–E), while CCR6 expressions were significantly decreased in CD8^+^ T cells (C01, C11, C17), CD4^+^ T cells (C03, C04, C14) NK cells (C07, C08), and NKT cells (C18) (Figures 2L–O and S4F–K). Importantly, we found that the expression levels of CCR6 decreased as the irAE grades increased (Figure 2L–O). The expression levels of CXCR3 and CCR6 in these subpopulations were more superior in predicting irAEs (Figure 2P,Q) and CCR6 expression in a Granzyme B (GZMB)^+^ CD57^low^ NK cell cluster completely differentiates patients with Grade 3 irAEs from those without irAEs (Figure 2R).

Since both CXCR3 and CCR6 were documented with roles in regulating the efficacy of immunotherapies [30, 34, 35], we hence divided the patients into PR and SD/PD group to evaluate the changes of CXCR3 and CCR6 in these subpopulations that differentiate irAE group and non_irAE group. Neither CXCR3 nor CCR6 was differentially expressed between PR and SD/PD group in these PBMC subpopulations (Figure S4L–U), suggesting irAE‐distinctive roles of CXCR3 and CCR6 in these subpopulations.

**FIGURE 3:**
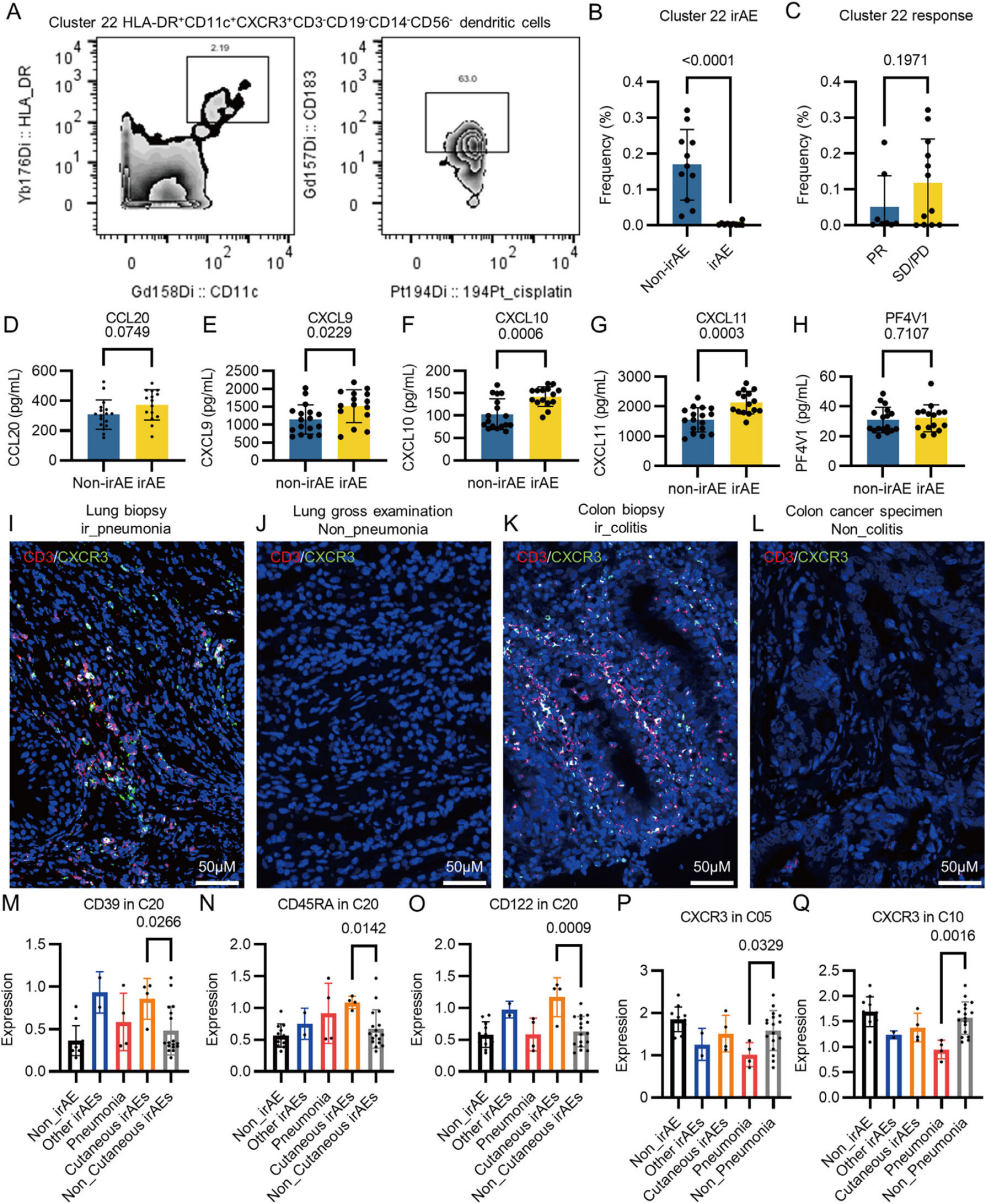
Circulating ligands of CXCR3 and CXCR3 expressing T cells in inflamed regions were elevated. (A) FACS gating of the Cluster 22. (B) The frequencies of CyTOF events for Clusters 22 among non_irAE, all grades irAE, Grade 1–2 irAE, and Grade 3 groups. (C) The frequencies of CyTOF events for Clusters 22 between PR and SD/PD groups. (D) Levels of plasma CCL20 of patients from irAE and non_irAE groups. (E) Levels of plasma CXCL9 of patients from irAE and non_irAE groups. (F) Levels of plasma CXCL10 of patients from irAE and non_irAE groups. (G) Levels of plasma CXCL11 of patients from irAE and non_irAE groups. (H) Levels of plasma PF4V1 of patients from irAE and non_irAE groups. (I) Multiple immunochemistry staining of CD3 and CXCR3 in the lung biopsy sample from a Grade 2 immune‐related pneumonia (G2_ir_pneumonia) patient. (J) Multiple immunochemistry staining of CD3 and CXCR3 in the lung gross examination sample from a Grade 2 immune‐related rash (G2_ir_rash) patient. (K) Multiple immunochemistry staining of CD3 and CXCR3 in the colon biopsy sample from a Grade 2 immune‐related colitis (G2_ir_pneumonia) patient. (L) Multiple immunochemistry staining of CD3 and CXCR3 in the colon cancer specimen from a Grade 2 immune‐related rash (G2_ir_rash) patient. (M) CD39 expression levels in C20 among non_irAE, pneumonia, cutaneous irAE, other irAE, and non_cutaneous irAE groups. (N) CD45RA expression levels in C20 among non_irAE, pneumonia, cutaneous irAE, other irAE, and non_cutaneous irAE groups. (O) CD122 expression levels in C20 among non_irAE, pneumonia, cutaneous irAE, other irAE, and non_cutaneous irAE groups. (P) CXCR3 expression levels in C05 among non_irAE, pneumonia, cutaneous irAE, other irAE, and non_pneumonia groups. (Q) CXCR3 expression levels in C10 among non_irAE, pneumonia, cutaneous irAE, other irAE, and non_pneumonia groups.

Apart from the divergences in marker expressions, a unique cluster (Cluster 22, HLA‐DR^+^CD11c^+^CXCR3^+^CD3^−^CD19^−^CD14^−^CD56^−^ cells) that is present only in patients without irAEs was identified (Figures 3A,B and S7), suggesting a potential role of these cells in preventing autoimmunity. The frequencies of this dendritic cell subpopulation showed no difference between PR and SD/PD group (Figure 3C), indicating its specialized antiautoimmunity role.

### 2.3 Circulating Ligands of CXCR3 and CXCR3 Expressing T Cells in Inflamed Regions Are Elevated

To further validate the participations of CXCR3 and CCR6 in PD‐1 inhibitor‐induced irAEs, we revisited the cohort and analyzed circulating CCL20, the ligand of CCR6, as well as CXCL9, CXCL10, CXCL11, and human platelet factor 4 variant (PF4V1), the ligands of CXCR3 (Figure 1A). Enzyme‐linked immunosorbent assay (ELISA) analysis revealed that baseline CXCL9, CXCL10, and CXCL11 (Figure 3E–G) but not CCL20 or PF4V1 (Figure 3D,H) were significantly elevated in the plasma in patients with irAEs, suggesting a baseline proinflammatory status associated with activated ligands–CXCR3 axis. To examine the involvement of CXCR3 in the adverse effects, CXCR3 was stained in situ in the irAE tissues as well as corresponding controls. We observed enrichments of CXCR3^+^CD3^+^ T cells in lung tissue with immune‐related pneumonia tissue (Figures 3I and S8A) as well as colon tissue with immune‐related colitis tissue (Figures 3K and S9A), while normal lung tissue (Figures 3J and S8B) and colon/colon adenocarcinoma tissue (Figures 3L and S9B) barely showed CXCR3^+^ cells. These data suggested that pre‐elevated CXCR3 ligands in circulation before ICI regimen later facilitate the infiltration of CXCR3‐positive T cells and promote the development of irAEs.

### 2.4 Biomarkers That Discriminate Specific irAEs

In the discovery cohort, four patients suffered from cutaneous irAEs, while another four patients suffered from pneumonia post ICI treatment (Table S1). We next tried to divide the patients into irAE‐specific group and tried to identify biomarkers that discriminate specific irAEs. We failed to identify biomarker at holistic PBMC level or PBMC subpopulation that discriminate specific irAE. Yet, we found one unique subpopulation (Cluster 20) in which the expressions of CD39, CD45RA, and CD122 are significantly increased in cutaneous irAE group in comparison with non_irAE group or non_cutaneous irAE group (Figure 3M–O). Meanwhile, we found that the expressions of CXCR3 in Cluster 05 and Cluster 10 are significantly decreased in pneumonia group in in comparison with non_irAE group or non_pneumonia irAE group (Figure 3P,Q). These data suggested the possibilities of identification of biomarkers that discriminate specific irAEs.

### 2.5 Validation of Decreased CXCR3 and CCR6 in PBMC Subpopulations with Two External Cohorts

**FIGURE 4:**
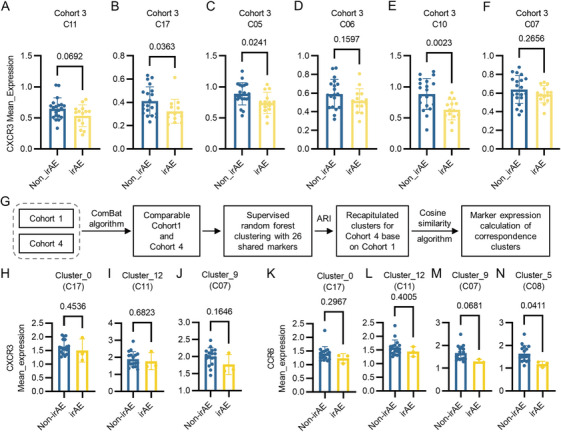
Validation of decreased CXCR3 and CCR6 in PBMC subpopulations in two external cohorts analyzed via CyTOF at baseline. (A) Expression levels of CXCR3 in Cluster 11 of validation Cohort 3 between irAE and non_irAE groups. (B) Expression levels of CXCR3 in Cluster 17 of validation Cohort 3 between irAE and non_irAE groups. (C) Expression levels of CXCR3 in Cluster 05 of validation Cohort 3 between irAE and non_irAE groups. (D) Expression levels of CXCR3 in Cluster 06 of validation Cohort 3 between irAE and non_irAE groups. (E) Expression levels of CXCR3 in Cluster 10 of validation Cohort 3 between irAE and non_irAE groups. (F) Expression levels of CXCR3 in Cluster 07 of validation Cohort 3 between irAE and non_irAE groups. (G) Flowchart illustrating recapitulation of clusters for Cohort 4 based on Cohort 1. (H) Expression levels of CXCR3 in Cluster 0 (Cluster 17 of Cohort 1) of validation Cohort 4 between irAE and non_irAE groups. (I) Expression levels of CXCR3 in Cluster 12 (Cluster 11 of Cohort 1) of validation Cohort 4 between irAE and non_irAE groups. (J) Expression levels of CXCR3 in Cluster 9 (Cluster 07 of Cohort 1) of validation Cohort 4 between irAE and non_irAE groups. (K) Expression levels of CCR6 in Cluster 0 (Cluster 17 of Cohort 1) of validation Cohort 4 between irAE and non_irAE groups. (L) Expression levels of CCR6 in Cluster 12 (Cluster 11 of Cohort 1) of validation Cohort 4 between irAE and non_irAE groups. (M) Expression levels of CCR6 in Cluster 9 (Cluster 07 of Cohort 1) of validation Cohort 4 between irAE and non_irAE groups. (N) Expression levels of CCR6 in Cluster 5 (Cluster 08 of Cohort 1) of validation Cohort 4 between irAE and non_irAE groups.

We next employed two external cohorts to validate the decrease of CXCR3 and CCR6 in identified PBMC subpopulations. The first external cohort (Cohort 3) consists of 34 esophageal squamous carcinoma patients that received PD‐1 inhibitors and 14 of them developed Grade 1–3 irAEs (Table S1). PBMCs were collected before the first PD‐1 inhibitor treatment and CyTOF was performed with same panel as Cohort 1 and the results were analyzed through a previously reported convolutional neural network method [36]. We found that the expressions of CXCR3 were decreased (Figure 4A–F) in clusters identified in Cohort 1 (Figure 2E–J). Among these subpopulations, the expressions of CXCR3 were significantly decreased in C17 (Figure 4B; CD57^+^CD27^−^CCR4^−^CD8^+^ T cell), C05 (Figure 4C; HLA‐DR^−^CD62L^low^CD4^+^ T cell), and C10 (Figure 4E; γδT cell). However, we did not observe the decreased CCR6 (Figure S10A–D) in previous identified clusters (Figure 2L‐O).

To further validate the decrease of CXCR3 and CCR6, another external cohort (Cohort 4) consists of 20 small cell lung cancer patients who received PD‐1 inhibitors was introduced. PBMCs were collected before the first PD‐1 inhibitor treatment and CyTOF was performed with a different panel that shared 26 markers with Cohort 1 (Table S2). ComBat algorithm was performed on the acrsinh transformed mass cytometry data and PCA (principal component analysis) was used to validate that the data of Cohort 1 and Cohort 4 are comparable (Figure 4G). A supervised random forest approach is employed, using the 26 shared markers from both cohorts to recapitulate the clustering results obtained with the 41 markers (Figure S11A) and a classification model containing 500 decision trees is trained, and the reconstruction effectiveness is evaluated using the adjusted rand index (ARI). To establish cross‐dataset cluster correspondence, the cosine similarity algorithm is used to calculate the similarity of cluster expression profiles between the two datasets (Figure S11B). Clustering distributions are intuitively compared through UMAP (Uniform Manifold Approximation and Projection) dimensionality reduction visualization (Figure S11C) and cluster heatmaps are generated to display expression patterns (Figure S11D). The expressions of both CXCR3 and CCR6 were decreased (Figure 4H–N) in recapitulated clusters corresponding to clusters identified in Cohort 1. Among them, the expression of CCR6 was significantly decreased in Cluster 5 of Cohort 4 (corresponding C08 in Cohort1), a CCR4^−^CD45RO^high^ NK cell population (Figure 4N). Taken together, our results were partially validated in two independent external cohorts with CyTOF data at baseline of ICI treatment demonstrating a high confidence potential of utilizing the expressions of CXCR3 and CCR6 in predicting ICI‐induced irAEs.

### 2.6 Identification of Differentially Expressed Protein Markers Between PR and SD/PD Group

**FIGURE 5:**
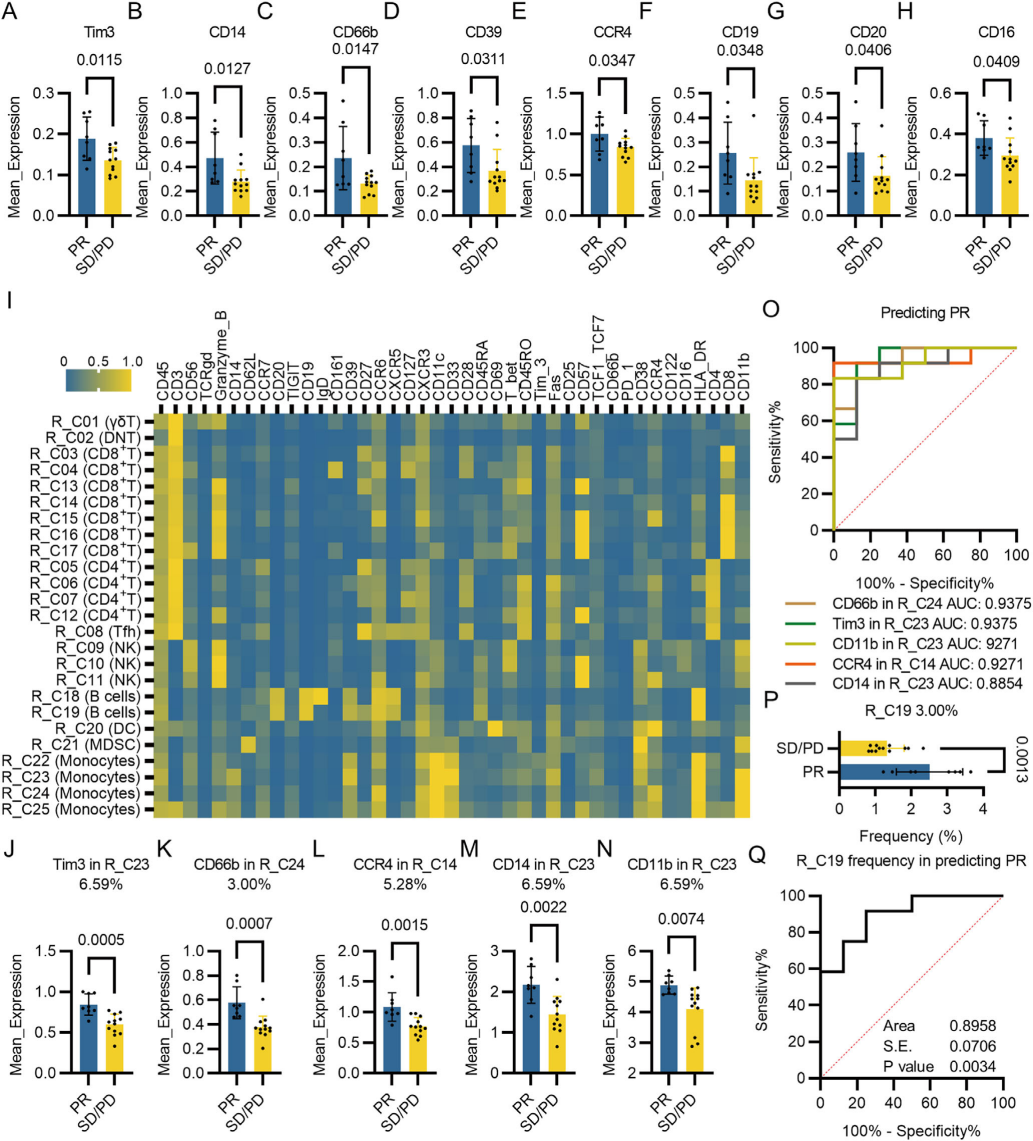
Identification of differentially expressed protein markers between PR and SD/PD group. (A) Tim3 expression levels in PR and SD/PD group. (B) CD14 expression levels in PR and SD/PD group. (C) CD66b expression levels in PR and SD/PD group. (D) CD39 expression levels in PR and SD/PD group. (E) CCR4 expression levels in PR and SD/PD group. (F) CD19 expression levels in PR and SD/PD group. (G) CD20 expression levels in PR and SD/PD group. (H) CD16 expression levels in PR and SD/PD group. (I) Heatmap illustrating 25 clusters identified based on the expressions of protein markers of 21 samples subjected to CyTOF. (J) Expression levels of Tim3 in Response Cluster 23 (R_C23) between PR and SD/PD groups. (K) Expression levels of CD66b in R_C24 between PR and SD/PD groups. (L) Expression levels of CCR4 in R_C14 between PR and SD/PD groups. (M) Expression levels of CD14 in R_C23 between PR and SD/PD groups. (N) Expression levels of CD11b in R_C23 between PR and SD/PD groups. (O) ROC curves and AUCs of CD66b in R_C24 (AUC: 0.9375, 95% CI: 0.8152–1.000, *p* value: 0.0012), Tim3 in R_C23 (AUC: 0.9375, 95% CI: 0.8262–1.000, *p* value: 0.0012), CD11b in R_C23 (AUC: 0.9271, 95% CI: 0.8132–1.000, *p* value: 0.0016), CCR4 in R_C14 (AUC: 0.9271, 95% CI: 0.8159–1.000, *p* value: 0.0016), and CD14 in R_C23 (AUC: 0.8854, 95% CI: 0.7335–1.000, *p* value: 0.0043) in predicting response to PD‐1 treatment. (P) Frequency of Response Cluster 19 (R_C19) in PBMCs from patients in PR or SD/PD groups. (Q) ROC curve of frequency of R_C19 (AUC: 0.8958, 95% CI: 0.7574–1.000, *p* value: 0.0034) in predicting response to PD‐1 treatment.

To excavate predictive biomarkers that authenticate the patients respond to PD‐1 inhibitor therapy before the treatment, CyTOF samples were divided into PR and SD/PD groups (Figure S12). Analysis of total PBMCs showed that expressions of Tim3, CD14, CD66b, CD39, CCR4, CD19, CD20, and CD16 were holistically decreased in the SD/PD group (Figure 5A–H). Among them, CD14 displayed best potency in predicting the response to PD‐1 inhibitor with an AUC of 0.8333 (Figure S13A). We further divided the PBMCs into 25 subpopulations via dimensionality reduction and clustering based on the protein expression levels of all 21 samples (Figure 5I). Greater discrepancies of protein levels between PR and SD/PD groups were identified in PBMC subpopulations (Figures 5J–N and S13B–K). The expressions of Tim‐3, CD14, and CD11b were all higher in a monocyte subpopulation, Response_Cluster23 (R_C23, “response cluster” were used to differentiate the clusters for irAE and non_irAE groups), of patients who responded well to PD‐1 inhibitor (Figure 5J,M,N). Meanwhile, CD66b expression was elevated in multiple PBMC subpopulations, especially in R_C24, another monocyte subpopulation (Figures 5K and S13F–K). Predication efficacies of these protein expression levels in PBMC subpopulations exceeded those in overall PBMCs (Figure 5O). Moreover, the frequencies of R_C19, a CCR6^+^CXCR5^+^ B cell subpopulation, were significantly higher in patients who responded well to PD‐1 inhibitor compared with those who did not respond to PD‐1 inhibitors (Figure 5P), suggesting the presence of a promising cell population in peripheral blood that can predict the efficacy of immunotherapy (Figure 5Q).

## 3 Discussion

**FIGURE 6:**
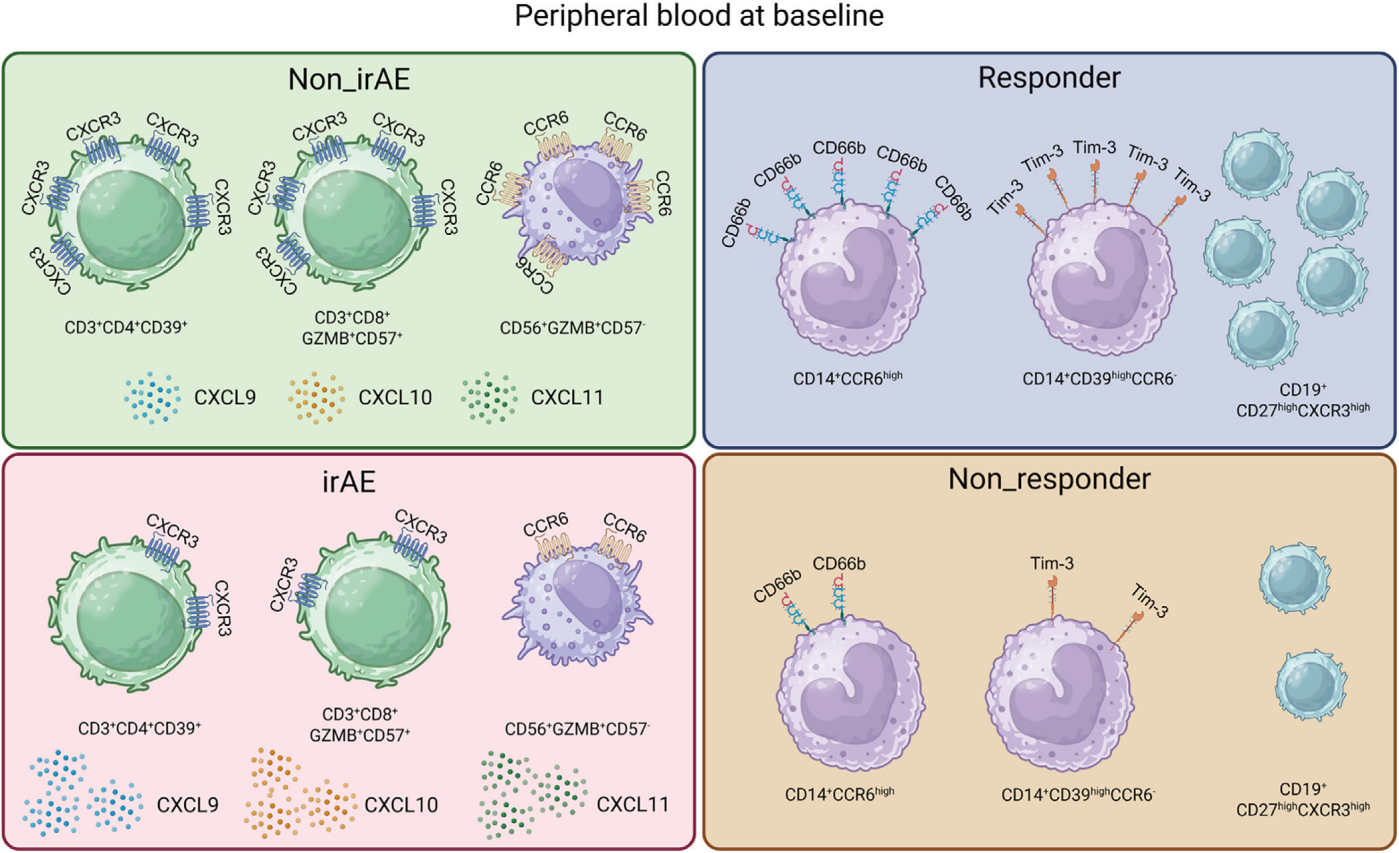
Illustration of PBMCs that predicts irAEs from and responses to PD‐1 inhibitor treatment.

With the rapid expansion of ICI utilizations in cancer treatments, the occurrence of irAEs and the resistance to ICIs are the two major challenges for clinicians and patients. Here, we employed CyTOF to decipher the critical subpopulations and protein expressions that may predict the occurrences of irAE as well as the resistances to PD‐1 inhibitors. Our results revealed a decreased baseline expression of CXCR3 in subpopulations of CD4^+^T, CD8^+^T, γδT, and NK cells in patients with irAEs. Baseline CCR6 expression was also decreased in subpopulations of CD8^+^T and NK cells in patients with irAEs. We also found that CD66b and Tim‐3 were significantly higher in subpopulations of monocytes in patients who responded to PD‐1 inhibitors. Notably, CD39, GZMB, and CD57 were critical in defining these subpopulations that predicts irAEs. The changed CXCR3 and CCR6 expressions in previous PBMC populations did not predict the outcome of PD‐1 inhibitor treatment indicating that the independency and complexity of PBMCs between inducing irAE and clearing tumor cells. However, we noticed that several PBMC populations defined by CXCR3 and CCR6 are of potential utilization in predicting the outcome of PD‐1 inhibitor treatment demonstrating the participants of CXCR3 and CCR6 in PD‐1 inhibitor‐based immunotherapy (Figure 6). Importantly, we have validated the decreases of CXCR3 and CCR6 in two independent cohorts consists of 34 esophageal squamous carcinoma patients and 20 small cell lung cancer. Even though the irAE of these two cohorts were milder than the identification cohort, we still observed significant decreases of CXCR3 in CD57^+^CD27^−^CCR4^−^CD8^+^ T cells, HLA‐DR^−^CD62L^low^CD4^+^ T cells, and γδT cells and decrease of CCR6 in CCR4^−^CD45RO^high^ NK cells. These multicenter data provided more confidence in utilizing the expressions of CXCR3 and CCR6 in predicting ICI‐induced irAEs. Additionally, frequency differences of PBMC subpopulations were also possible to predict the occurrences of irAE and the resistance to PD‐1 inhibitors, providing easier tools for further clinical applications of ICIs.

Based on these findings, an overall scoring system could be established by combining the frequency of Cluster 22, the CCR6 expression in C07, and the frequency of Response Cluster 19 to evaluate both prognosis and toxicity of ICI treatment. When the frequency of Response Cluster 19 is greater than 1.219, two points are scored, when the frequency of Cluster 22 is greater than 0.025, one point is scored and when the CCR6 expression in C07 is greater than 1.238, another one point is scored. Based on this scoring system, when two or more points is scored by a patient before the application of ICI regimen, the regimen is considered beneficial. Otherwise, the regimen is discouraging.

Prediction of irAEs resulted from ICI has long been a concern for both clinicians and patients and tremendous effects have been put in examining the circulating biomolecules and cells due to the convenience of blood samples [14, 37, 38]. In our cohort, baseline CXCR3 and CCR6 were decreased in PBMCs from patients who developed irAE after PD‐1 inhibitor treatment, but the circulating expressions of CXCL9, CXCL10, and CXCL11 were increased. More importantly, irAE in situ tissue exhibited enrichments of CXCR3^+^ cells. CXCR3 expression has initially been reported to be associated with increased efficacy with anti‐PD‐1 treatment [30]. CyTOF has revealed that a CXCR3^+^CD8^+^ effector memory T cell population was decreased in pretreatment samples from HCC patients who developed irAEs after PD‐1 inhibitor treatments [35]. Similarly, scRNA‐seq profiling of PBMCs showed that CCR6^+^CXCR3^+^CD8^+^ T cells were decreased in rheumatic irAE patient in comparison with rheumatoid arthritis control patients or healthy individuals [39]. In contrast, a CXCR3^hi^ CD8^+^ T subpopulation was found enriched in the synovial fluid samples from PD‐1 inhibitor‐treated patients with arthritis‐irAE [40] and another analysis showed that the percentage of CXCR3^+^ CD4^+^ Tcm cells and CXCR3^+^ CD8^+^ TemRA cells were increased at baseline in patients who developed irAE after receiving ICI treatment [23]. The role CXCR3 plays during irAEs requires more elaborate analysis of the irAE tissues and, more directly, animal models. It has been shown that PD‐1 inhibitor treatment has induced the percentage of CXCR3^+^ T cells but a sustained high percentage of CXCR3^+^ T cells is corelated with PD [41]. Further study revealed that infiltration of CXCR3^+^ Treg sabotaged anti‐PD‐1 immunotherapy as an immune suppressor [42]. As for the ligands, CXCL9 and CXCL10 were shown to facilitate PD‐1 inhibitor treatments [41] via promote CD8^+^ T‐cell infiltration [43]. Moreover, CXCR3/CXCL9 axis was found critical for IL‐12 antitumor efficacy to benefit to anti‐PD‐1 therapies [44] and in an ICI myocarditis mice model, scRNA‐seq revealed macrophages with high level *Cxcl9* and *Cxcl10*, two genes encoding CXCR3 ligands, expression levels were increased [45]. These results demonstrated the involvement of chemokine–CXCR3 axis in ICI‐induced irAEs, yet, how to manipulate the dual roles of CXCR3 expressing T cells to promote the efficacy of PD‐1 inhibitor therapy and preventing irAEs at the same time requires refined analysis. Our results revealed limited information related to the changes of frequencies of these T cell subpopulations between irAE and non_irAE groups. However, a CXCR3‐positive dendritic cells were absent in irAE groups indicating that these circulating dendritic cells are protecting the organs from PD‐1 antibody‐induced damage. CXCR3‐deficient mice and CXCR3‐neutralizing antibody are powerful tools in defining the bona fide role CXCR3 plays in PD‐1 inhibitor‐induced irAEs. Yet, CXCR3 might play a more complex role than an immuno‐suppressor due to the complexity of immune cells. Immunosuppression effect was observed for CCL20–CCR6 axis [46], which is in line with our result that higher CCR6 expressions in PBMCs are correlated to decreased irAE risks. Together with these published data, our results revealed that CXCR3 and CCR6 are indispensable for immunotherapy. Yet, in‐depth analysis of the heterogeneity of immune cell constitutions and status are vital for monitoring and manipulating CXCR3 and CCR6 during immunotherapy.

PD‐L1 protein levels, tumor mutation burden, and microsatellite‐instability are currently United States Food and Drug Administration‐approved biomarkers that are of substantial value and limitations [47]. The heterogenicity of tumors and immune systems of individuals urge for novel and more biomarkers that indicates responses to immunotherapies. Recent advancements have provided new tools in predicting immunotherapy outcomes as well as expended the populations that may benefit from immunotherapy [48, 49, 50]. Among them, immune cell profiling provided insights to enhance clinical outcomes while minimizing risks [51, 52]. Our data suggested a CXCR3^high^ B cell population is in favor of clinical outcomes and the expressions of CD66b and Tim‐3 in monocyte subpopulations are correlated to responses to PD‐1 inhibitors. Importantly, biomarkers identified for irAE and response were independent.

A vital limitation of our study is the limited sample size of the identification cohort due to difficulties in harvesting and preserving high quality PBMCs and in evaluating irAEs. Larger cohorts focusing on individual cancer type might validate the markers identified and bring to light to undiscovered markers in organ‐specific irAEs, early‐onset and late‐onset irAEs, or multiple irAEs [53, 54, 55]. Although a scoring system for the prediction of irAEs was established, multiomics biomarkers integrating CyTOF and clinical features can more accurately predict the occurrence of irAEs. Prospective validations are also needed before these markers are ready for clinical applications. Meanwhile, a dynamic analysis of PBMCs would provide more intact information on the role of CXCR3 and CCR6 during the entire process of immunotherapy. Conditional knockout of either gene in specific PBMC subpopulations would provide not only experimental evidence of the involvements of CXCR3 and CCR6 in ICI‐induced irAEs but proof of concept possibility of CXCR3 and CCR6 manipulation to prevent irAEs. Nonetheless, our study has demonstrated the possible use of CyTOF to identify biomarkers for predicting the occurrences of irAEs and the resistances to PD‐1 inhibitor treatment. This protein‐based and membrane‐protein‐preference method is easier for translational medicine since it is easier and faster in comparison with scRNA‐seq. With a solid and prospective validation in future, these identified biomarkers are of significant values in the molecular toolbox of immunotherapy.

## 4 Conclusion

In patients who developed irAEs after PD‐1 inhibitor treatment, CXCR3 and CCR6 were significantly decreased in PBMC and PBMC subpopulations. The expression of CCR6 in a NK cell subpopulation as well as the percentage of a circulating CXCR3^+^ dendritic cell subpopulation serves as excellent biomarkers in predicting PD‐1 inhibitor‐induced irAE independent of the responses to PD‐1 inhibitor. Importantly, we found that CXCR3^+^ T cells were enriched in irAE tissues. This work has further sculptured the landscape of immune cell heterogeneity via focusing on the roles of CXCR3 and CCR6‐positive PBMCs and provides refined biomarkers to predict the occurrences of irAEs and the resistances to PD‐1 antibodies.

## 5 Materials and Methods

### 5.1 Patient Cohorts

This study was approved by the Ethics Committee of the First Affiliated Hospital of Shandong First Medical University (No. YXLL‐KY‐2022–059), the Ethics Committee of the First Medical Center of Chinese PLA General Hospital (S2022‐170‐02), and the Ethics Committee of the Hunan Cancer Hospital (KY2002232). All patients were informed and signed the consent forms and all experiments were conducted in accordance with the principles of the Declaration of Helsinki.

Patient Cohort 1 (discovery cohort) consists of 32 pan‐cancer patients who received PD‐1 inhibitors (camrelizumab, pembrolizumab, sintilimab, tislelizumab, or toripalimab) alone or in combination with chemotherapy or targeted therapy in the First Affiliated Hospital of Shandong First Medical University March 2020 to November 2023 (Tables 1 and S1). PBMCs and plasma were collected 1 day before the PD‐1 inhibitor treatment. Treatment response was assessed as complete response, PR, SD, and PD according to RECIST 1.1 after two cycles of PD‐1 inhibitor treatments. The irAEs were graded according to the National Cancer Institute Common Terminology Criteria for Adverse Events, version 5.0. PD‐1 inhibitor‐induced irAEs were initially diagnosed by medical oncologist (H.X, S.L, Y.G, and J.W) and subsequently confirmed by a multidisciplinary team at the First Affiliated Hospital of Shandong First Medical University. All patients who received at least two cycles of ICI therapy and irAEs were monitored for at least 14 months after ICI therapy although ICI could be discontinued, unless the patient died. Patients who developed one or multiple irAEs following ICI therapy were defined as “irAE group,” and those who did not develop any irAEs following ICI therapy were defined as “non_irAE group.” Here, we only focused on the most severe irAE in one patient if multiple irAEs coexisted.

Patient Cohort 2 (validation cohort) consists of two patients with lung adenocarcinoma, one with stomach adenocarcinoma, and one with colon cancer. The lung biopsy was acquired from a lung adenocarcinoma patient suspicious of metastasis, while the lung gross examination was acquired from the other lung adenocarcinoma patient during surgery. The colon biopsy was acquired from a stomach adenocarcinoma patient suspicious of metastasis, while the colon cancer specimen was acquired from the patient with colon cancer during surgery.

Patient Cohort 3 (validation cohort) consists of 34 esophageal squamous carcinoma patients received PD‐1 inhibitors (pembrolizumab, sintilimab, or toripalimab) in First Medical Center of Chinese PLA General Hospital and Patient Cohort 4 (validation cohort) consists of 20 small cell lung cancer patients received PD‐1 inhibitors (durvalumab or atezolizumab) in Hunan Cancer Hospital. All PBMCs were collected 1 day before the PD‐1 inhibitor treatment and detailed information is in Table S1. The irAEs were graded according to the National Cancer Institute Common Terminology Criteria for Adverse Events, version 5.0 by multidisciplinary teams in corresponding hospitals.

### 5.2 Cytometry by Time of Flight

Eight milliliters of peripheral blood from each patient were collected 1 day before the treatment with PD‐1 inhibitor and processed within 24 h. PBMCs were isolated density gradient centrifugation using Ficoll before cryopreserved in liquid nitrogen. Before mass cytometry staining, cells were thawed and resuspended in precooled 1 × buffer (phosphate buffered saline supplemented with 0.5% bovine serum albumin). PBMCs were washed once with 1 × buffer and the number and viability of cells were measured. The number of cells should be no less than 3 × 10^6^ and the viability rate should be higher than 85% before antibody staining. A complete antibody and metal tag lists are available in Table S2. Antibody labeling was performed using the MaxPAR antibody Labelling kit (Fluidigm). Mass cytometry was performed as previously described [56]. Briefly, cells were washed and stained cisplatin (Fluidigm) to exclude dead cells. Cells were incubated in Fc receptor blocking solution and stained with surface antibodies for 30 min on ice. Cells were washed twice with 1× buffer and fixed intercalation solution (Maxpar Fix and Perm Buffer containing 250 nM 191/193Ir, Fluidigm) overnight. After fixation, cells were washed and stained with intracellular antibodies for 30 min on ice. Cells were washed and resuspend with deionized water with 20% EQ beads (Fluidigm) and mass cytometry was performed on a mass cytometer (Helios, Fluidigm).

### 5.3 CyTOF Data Analysis

Data were first debarcoded from raw data and normalized through bead normalization method [57, 58]. The cyCombine algorithms was introduced to minimalize batch effects [59]. FlowJo software (V10.8.1) was used for gating to exclude to debris, dead cells, and doublets resulting in live, single immune cells. X‐shift clustering algorithm was employed to distinct phenotypes based on marker expression levels and *t*‐SNE algorithm was employed to reduce dimensionality and reveal distribution of each cluster and marker expression and difference among each group or different sample type. Clusters were annotated manually based on the expression levels of symbolic markers [60, 61].

### 5.4 Cross‐Dataset Immune Cell Subpopulation Identification and Validation

The mass cytometry data from two cohorts (Cohort 1 and Cohort 4) are first read. After excluding technical parameter channels, arcsinh transformation is applied to 27 shared immune markers to approximate a normal distribution, and batch correction is performed using the ComBat algorithm [62]. The correction effect is validated via PCA and distribution plots to ensure data comparability. Subsequently, unsupervised analysis is conducted on Cohort 1 using the PARC clustering algorithm. The original Cohort 1 data, comprising 41 original markers, is subjected to clustering analysis. Dimensionality reduction and downsampling are performed, selecting 100,000 cells per group. The target number of clusters is set to 26, and the algorithm automatically selects the optimal resolution parameter between 0.5 and 3.0 with a step size of 0.02. Next, a supervised random forest approach is employed, using the 27 shared markers from both cohorts to recapitulate the clustering results obtained with the 41 markers. A classification model containing 500 decision trees is trained, and the reconstruction effectiveness is evaluated using the ARI. Clustering distributions are intuitively compared through UMAP dimensionality reduction visualization, and cluster heatmaps are generated to display expression patterns. A binary random forest model is trained for each cell subpopulation in Cohort 1. Marker contribution is assessed using permutation importance, and model performance is verified using metrics such as accuracy and sensitivity. The trained models are then applied to Cohort 4 for independent validation, predicting cell assignment probabilities. To establish cross‐dataset cluster correspondence, the cosine similarity algorithm is used to calculate the similarity of cluster expression profiles between the two datasets. By comparing the vector directions of the *Z*‐score normalized expression matrices, the best‐matching clusters are identified. Finally, high‐confidence cells are filtered based on a prediction probability threshold (>0.8). The average expression of the 26 markers within each sample is calculated, generating standardized output containing an expression matrix and cell counts.

### 5.5 Enzyme‐Linked Immunosorbent Assay

For each sample, 50 µL plasma was collected before the treatment with PD‐1 inhibitors and used for ELISA. ELISA was performed according to the manufacturer's instructions: human MIP‐3 alpha ELISA kit (Abcam; ab269562), human CXCL11 ELISA kit (Abcam; ab289695), human IP‐10 ELISA kit (Abcam; ab83700), human CXCL9 ELISA kit (Abcam; ab219047), and PF4V1 ELISA kit (CUSABIO; CSB‐EL017810HU).

### 5.6 Multiplex Immunofluorescence

Paraffin embedded tissue sections were deparaffinized and rehydrated with xylene and alcohol. Epitope retrieval was performed with microwave boiling in sodium citrate buffer. Anti‐CD3 antibody (Abcam; ab16669), anti‐CD183 antibody (Abcam; ab288437), and anti‐CD196 antibody (Abcam; ab227036) and corresponding secondary antibodies were sequentially incubated with the sections. DAPI was used to visualize nucleus. TissueFAXS (TissueGnostics) with a Zeiss Axio Imager Z2 Microscope System at ×20 magnification was applied to acquire the images.

### 5.7 Statistical Analysis

Results are presented as means ± S.D., and statistical analyses were performed in Graphpad version 9.0.0 using two tailed Student's *t*‐test for two groups. The *p* < 0.05 was considered significant. 95% confidence interval and Wilson/Brown method were used for ROC calculation. Multiple logistic regression was performed to establish bivariant models for predictions.

## Author Contributions

Jun Wang: conceptualization, data curation, methodology, investigation, writing – original draft, writing – review and editing, funding, and project administration. Hong Xie: conceptualization, data curation, and methodology. Yuanyuan Gong: bioinformatics, data curation, and formal analysis. Songlin Liu: data curation and methodology. Yaping Guan: data curation and methodology. Yuekai Zhang: data curation and methodology. Qi Xie: data curation and methodology. Jingyi Wang: data curation and resources. Ye Li: data curation and resources. Xueqin Zeng: pathological analysis. Xi Chen: conceptualization, writing – original draft, writing – review and editing, and funding. Chen Wang: conceptualization, data curation, methodology, formal analysis, investigation, writing – original draft, writing – review and editing, funding, and project administration. All authors have read and approved the final manuscript.

## Funding

Chen Wang and Yuanyuan Gong was supported by the construction project of Shanghai Key Laboratory of Molecular Imaging (18DZ2260400) and the ShanghaiTech University (2013F0301‐000‐06). Xi Chen was supported by The Discipline Construction Projects of Guangzhou Medical University (02‐412‐2302‐2178XM) of the Guangzhou Medical University. This work was partially supported by Natural Science Foundation of Shandong Province (Grant No. ZR2021MH291), Cultivating Fund of The First Affiliated Hospital of Shandong First Medical University (Grant No. QYPY2022NSFC0613), CSCO‐MSD Cancer Research Foundation (Grant No. Y‐MSD2020‐0350), CSCO‐PILOT Cancer Research Foundation (Grant No. Y‐2019AZMS‐0440), and Wu Jieping Medical Foundation for Clinical Scientific Research (Grant No. 320.6750.2020‐12‐16). The funders played no role in the study design, data collection and analysis, decision to publish, or preparation of the manuscript.

## Ethics Statement

This study complied with the ethical guidelines established by the Declaration of Helsinki. Ethical approval was obtained from the Research Ethics Committee of the First Affiliated Hospital of Shandong First Medical University (No. YXLL‐KY‐2022–059), the Ethics Committee of the First Medical Center of Chinese PLA General Hospital (S2022‐170‐02), and the Ethics Committee of the Hunan Cancer Hospital (KY2002232). All patients signed informed consent forms. The authors confirm that all the methods were performed in accordance with the relevant guidelines.

## Conflicts of Interest

The authors declare no conflicts of interest.

## Supporting information




**Supporting Figure 1**: Protein markers expression levels between irAE and non_irAE group.
**Supporting Figure 2**: TSNE plot illustrating the differential levels and distributions of 41 markers respectively.
**Supporting Figure 3**: The frequencies of CyTOF events for each clusters between irAE and non_irAE group.
**Supporting Figure 4**: CXCR3 and CCR6 expression levels in PBMC subpopulations between irAE and non_irAE group and their expression in corresponding PBMC subpopulations between PR and SD/PD group.
**Supporting Figure 5**: PD‐1 and TIGIT expressions in PBMC subpopulations between irAE and non_irAE groups.
**Supporting Figure 6**: Fas and CD25 expressions in PBMC subpopulations between irAE and non_irAE groups.
**Supporting Figure 7**: Gating of Cluster 22.
**Supporting Figure 8**: Full image of lung biopsy and partial image of lung gross examination results at same magnification.
**Supporting Figure 9**: Full image of colon biopsy and partial image of colon cancer specimen results at same magnification.
**Supporting Figure 10**: CCR6 expression in PBMC subpopulations of Cohort 3.
**Supporting Figure 11**: Recapitulating clusters for Cohort 4 base on Cohort 1.
**Supporting Figure 12**: Identification of differentially expressed protein markers between PR and SD/PD group.
**Supporting Figure 13**: Identification of differentially expressed protein markers between PR and SD/PD group in PBMC subpopulations.
**Supporting Table 1**: Detailed patient information of cohort 1 and cohort 2.
**Supporting Table 1 (continued)**: Detailed patient information of cohort 3 and cohort 4.
**Supporting Table 2**: Iron channels and antibodies for CyTOF.

## Data Availability

The data that support the findings of this study are available from the corresponding author, C.W., upon reasonable request.

## References

[1] R. D. Schreiber , L. J. Old , and M. J. Smyth , “Cancer Immunoediting: Integrating Immunity's Roles in Cancer Suppression and Promotion,” Science 331, no. 6024 (2011): 1565–1570. 21436444 10.1126/science.1203486

[2] Y. Ishida , Y. Agata , K. Shibahara , and T. Honjo , “Induced Expression of PD‐1, a Novel Member of the Immunoglobulin Gene Superfamily, Upon Programmed Cell Death,” Embo Journal 11, no. 11 (1992): 3887–3895. 1396582 10.1002/j.1460-2075.1992.tb05481.x PMC556898

[3] Y. Iwai , M. Ishida , Y. Tanaka , T. Okazaki , T. Honjo , and N. Minato , “Involvement of PD‐L1 on Tumor Cells in the Escape from Host Immune System and Tumor Immunotherapy by PD‐L1 Blockade,” Proceedings of the National Academy of Sciences of the United States of America 99, no. 19 (2002): 12293–12297. 12218188 10.1073/pnas.192461099 PMC129438

[4] H. Dong , S. E. Strome , D. R. Salomao , et al., “Tumor‐Associated B7‐H1 Promotes T‐cell Apoptosis: A Potential Mechanism of Immune Evasion,” Nature Medicine 8, no. 8 (2002): 793–800. 10.1038/nm730 12091876

[5] H. Nishimura , M. Nose , H. Hiai , N. Minato , and T. Honjo , “Development of Lupus‐Like Autoimmune Diseases by Disruption of the PD‐1 Gene Encoding an ITIM Motif‐Carrying Immunoreceptor,” Immunity 11, no. 2 (1999): 141–151. 10485649 10.1016/s1074-7613(00)80089-8

[6] H. Nishimura , T. Okazaki , Y. Tanaka , et al., “Autoimmune Dilated Cardiomyopathy in PD‐1 Receptor‐Deficient Mice,” Science 291, no. 5502 (2001): 319–322. 11209085 10.1126/science.291.5502.319

[7] P. Gougis , F. Jochum , B. Abbar , et al., “Clinical Spectrum and Evolution of Immune‐Checkpoint Inhibitors Toxicities over a Decade‐a Worldwide Perspective,” EClinicalMedicine 70 (2024): 102536. 38560659 10.1016/j.eclinm.2024.102536 PMC10981010

[8] S. M. Blum , D. A. Zlotoff , N. P. Smith , et al., “Immune Responses in Checkpoint Myocarditis across Heart, Blood and Tumour,” Nature 636, no. 8041 (2024): 215–223. 39506125 10.1038/s41586-024-08105-5 PMC12952943

[9] M. F. Thomas , K. Slowikowski , K. Manakongtreecheep , et al., “Single‐Cell Transcriptomic Analyses Reveal Distinct Immune Cell Contributions to Epithelial Barrier Dysfunction in Checkpoint Inhibitor Colitis,” Nature Medicine 30, no. 5 (2024): 1349–1362. 10.1038/s41591-024-02895-x PMC11673812 38724705

[10] M. Damo , N. I. Hornick , A. Venkat , et al., “PD‐1 Maintains CD8 T Cell Tolerance Towards Cutaneous Neoantigens,” Nature 619, no. 7968 (2023): 151–159. 37344588 10.1038/s41586-023-06217-y PMC10989189

[11] Y. Q. Gao , Y. J. Tan , and J. Y. Fang , “Roles of the Gut Microbiota in Immune‐Related Adverse Events: Mechanisms and Therapeutic Intervention,” Nature Reviews Clinical Oncology 22, no. 7 (2025): 499–516. 10.1038/s41571-025-01026-w 40369317

[12] S. Groha , S. A. Alaiwi , W. Xu , et al., “Germline Variants Associated with Toxicity to Immune Checkpoint Blockade,” Nature Medicine 28, no. 12 (2022): 2584–2591. 10.1038/s41591-022-02094-6 PMC10958775 36526723

[13] K. Esfahani , A. Elkrief , C. Calabrese , et al., “Moving Towards Personalized Treatments of Immune‐Related Adverse Events,” Nature Reviews Clinical Oncology 17, no. 8 (2020): 504–515. 10.1038/s41571-020-0352-8 32246128

[14] Y. Xu , Y. Fu , B. Zhu , J. Wang , and B. Zhang , “Predictive Biomarkers of Immune Checkpoint Inhibitors‐Related Toxicities,” Frontiers in Immunology 11 (2020): 2023. 33123120 10.3389/fimmu.2020.02023 PMC7572846

[15] R. J. Sullivan , A. R. Cillo , R. L. Ferris , et al., “SITC Vision: Opportunities for Deeper Understanding of Mechanisms of Anti‐Tumor Activity, Toxicity, and Resistance to Optimize Cancer Immunotherapy,” Journal for ImmunoTherapy of Cancer 13, no. 6 (2025): e011929. 40562704 10.1136/jitc-2025-011929 PMC12198810

[16] C. J. Kao , S. Charmsaz , S. L. Alden , et al., “Immune‐Related Events in Individuals with Solid Tumors on Immunotherapy Associate with Th17 and Th2 Signatures,” Journal of Clinical Investigation 134, no. 20 (2024): e176567. 39403935 10.1172/JCI176567 PMC11473156

[17] E. Papalexi and R. Satija , “Single‐Cell RNA Sequencing to Explore Immune Cell Heterogeneity,” Nature Reviews Immunology 18, no. 1 (2018): 35–45. 10.1038/nri.2017.76 28787399

[18] S. C. Bendall , E. F. Simonds , P. Qiu , et al., “Single‐Cell Mass Cytometry of Differential Immune and Drug Responses Across a Human Hematopoietic Continuum,” Science 332, no. 6030 (2011): 687–696. 21551058 10.1126/science.1198704 PMC3273988

[19] N. G. Steele , E. S. Carpenter , S. B. Kemp , et al., “Multimodal Mapping of the Tumor and Peripheral Blood Immune Landscape in Human Pancreatic Cancer,” Nature Cancer 1, no. 11 (2020): 1097–1112. 34296197 10.1038/s43018-020-00121-4 PMC8294470

[20] J. G. Castillo , R. DeBarge , A. Mende , et al., “A Mass Cytometry Approach to Track the Evolution of T Cell Responses during Infection and Immunotherapy by Paired T Cell Receptor Repertoire and T Cell Differentiation State Analysis,” BioRxiv (2024).

[21] M. M. Gubin , E. Esaulova , J. P. Ward , et al., “High‐Dimensional Analysis Delineates Myeloid and Lymphoid Compartment Remodeling During Successful Immune‐Checkpoint Cancer Therapy,” Cell 175, no. 4 (2018): 1014–1030.e19. 30343900 10.1016/j.cell.2018.09.030 PMC6501221

[22] Q. Zhang , M. Wang , Y. Li , et al., “Efficacy, Safety and Exploratory Analysis of Neoadjuvant Tislelizumab (A PD‐1 Inhibitor) plus Nab‐Paclitaxel Followed by Epirubicin/Cyclophosphamide for Triple‐Negative Breast Cancer: A Phase 2 TREND Trial,” Signal Transduction and Targeted Therapy 10, no. 1 (2025): 169. 40414961 10.1038/s41392-025-02254-3 PMC12104340

[23] Y. Qi , H. Ge , X. Sun , et al., “Systemic Immune Characteristics Predicting Toxicity to Immune Checkpoint Inhibitors in Patients with Advanced Breast Cancer,” Journal of Autoimmunity 153 (2025): 103423. 40267835 10.1016/j.jaut.2025.103423

[24] Y. Weng , S. J. Siciliano , K. E. Waldburger , et al., “Binding and Functional Properties of Recombinant and Endogenous CXCR3 Chemokine Receptors,” Journal of Biological Chemistry 273, no. 29 (1998): 18288–18291. 9660793 10.1074/jbc.273.29.18288

[25] C. Sgadari , J. M. Farber , A. L. Angiolillo , et al., “Mig, the Monokine Induced by Interferon‐Gamma, Promotes Tumor Necrosis in Vivo,” Blood 89, no. 8 (1997): 2635–2643. 9108380

[26] K. Hieshima , T. Imai , G. Opdenakker , et al., “Molecular Cloning of a Novel Human CC Chemokine Liver and Activation‐Regulated Chemokine (LARC) Expressed in Liver. Chemotactic Activity for Lymphocytes and Gene Localization on Chromosome 2,” Journal of Biological Chemistry 272, no. 9 (1997): 5846–5853. 9038201 10.1074/jbc.272.9.5846

[27] M. Baba , T. Imai , M. Nishimura , et al., “Identification of CCR6, the Specific Receptor for a Novel Lymphocyte‐Directed CC Chemokine LARC,” Journal of Biological Chemistry 272, no. 23 (1997): 14893–14898. 9169459 10.1074/jbc.272.23.14893

[28] W. W. Hancock , B. Lu , W. Gao , et al., “Requirement of the Chemokine Receptor CXCR3 for Acute Allograft Rejection,” Journal of Experimental Medicine 192, no. 10 (2000): 1515–1520. 11085753 10.1084/jem.192.10.1515 PMC2193193

[29] H. D. Hickman , G. V. Reynoso , B. F. Ngudiankama , et al., “CXCR3 Chemokine Receptor Enables Local CD8(+) T Cell Migration for the Destruction of Virus‐Infected Cells,” Immunity 42, no. 3 (2015): 524–537. 25769612 10.1016/j.immuni.2015.02.009 PMC4365427

[30] M. T. Chow , A. J. Ozga , R. L. Servis , et al., “Intratumoral Activity of the CXCR3 Chemokine System Is Required for the Efficacy of Anti‐PD‐1 Therapy,” Immunity 50, no. 6 (2019): 1498–1512.e5. 31097342 10.1016/j.immuni.2019.04.010 PMC6527362

[31] R. Varona , V. Cadenas , L. Gomez , A. C. Martinez , and G. Marquez , “CCR6 Regulates CD4+ T‐Cell‐Mediated Acute Graft‐Versus‐Host Disease Responses,” Blood 106, no. 1 (2005): 18–26. 15774622 10.1182/blood-2004-08-2996

[32] M. Bonelli , A. Puchner , L. Goschl , et al., “CCR6 controls Autoimmune but Not Innate Immunity‐Driven Experimental Arthritis,” Journal of Cellular and Molecular Medicine 22, no. 11 (2018): 5278–5285. 30133119 10.1111/jcmm.13783 PMC6201376

[33] Y. Lu , S. Hong , H. Li , et al., “Th9 cells Promote Antitumor Immune Responses in Vivo,” Journal of Clinical Investigation 122, no. 11 (2012): 4160–4171. 23064366 10.1172/JCI65459 PMC3484462

[34] H. Kagamu , S. Yamasaki , S. Kitano , et al., “Single‐Cell Analysis Reveals a CD4+ T‐Cell Cluster That Correlates with PD‐1 Blockade Efficacy,” Cancer Research 82, no. 24 (2022): 4641–4653. 36219677 10.1158/0008-5472.CAN-22-0112 PMC9755963

[35] S. Chuah , J. Lee , Y. Song , et al., “Uncoupling Immune Trajectories of Response and Adverse Events from Anti‐PD‐1 Immunotherapy in Hepatocellular Carcinoma,” Journal of Hepatology 77, no. 3 (2022): 683–694. 35430299 10.1016/j.jhep.2022.03.039

[36] Z. Hu , A. Tang , J. Singh , S. Bhattacharya , and A. J. Butte , “A Robust and Interpretable End‐to‐End Deep Learning Model for Cytometry Data,” Proceedings of the National Academy of Sciences of the United States of America 117, no. 35 (2020): 21373–21380. 32801215 10.1073/pnas.2003026117 PMC7474669

[37] B. Ponvilawan , A. W. Khan , J. Subramanian , and D. Bansal , “Non‐Invasive Predictive Biomarkers for Immune‐Related Adverse Events due to Immune Checkpoint Inhibitors,” Cancers (Basel) 16, no. 6 (2024): 1225. 38539558 10.3390/cancers16061225 PMC10968874

[38] S. Liu , Y. Zhang , Y. Guan , et al., “Risk Factors and Outcomes for Steroid‐Refractory Immune‐Related Hepatotoxicity in Locally Advanced and Metastatic Cancer,” Cancer Immunology, Immunotherapy 75, no. 2 (2026): 43. 41591499 10.1007/s00262-025-04287-7 PMC12847509

[39] X. Zhu , Y. Yu , Y. Li , et al., “Inflammatory Arthritis Immune Related Adverse Events Represent a Unique Autoimmune Disease Entity Primarily Driven by T Cells, but Likely Not Autoantibodies,” medRxiv (2025).

[40] S. T. Kim , Y. Chu , M. Misoi , et al., “Distinct Molecular and Immune Hallmarks of Inflammatory Arthritis Induced by Immune Checkpoint Inhibitors for Cancer Therapy,” Nature Communications 13, no. 1 (2022): 1970. 10.1038/s41467-022-29539-3 PMC9005525 35413951

[41] X. Han , Y. Wang , J. Sun , et al., “Role of CXCR3 Signaling in Response to Anti‐PD‐1 Therapy,” EBioMedicine 48 (2019): 169–177. 31521609 10.1016/j.ebiom.2019.08.067 PMC6838359

[42] M. A. Moreno Ayala , T. F. Campbell , and C. Zhang , “CXCR3 Expression in Regulatory T Cells Drives Interactions With Type I Dendritic Cells in Tumors to Restrict CD8(+) T Cell Antitumor Immunity,” Immunity 56, no. 7 (2023): 1613–1630.e5. 37392735 10.1016/j.immuni.2023.06.003 PMC10752240

[43] I. G. House , P. Savas , J. Lai , et al., “Macrophage‐Derived CXCL9 and CXCL10 Are Required for Antitumor Immune Responses Following Immune Checkpoint Blockade,” Clinical Cancer Research 26, no. 2 (2020): 487–504. 31636098 10.1158/1078-0432.CCR-19-1868

[44] J. Y. Lee , B. Nguyen , A. Mukhopadhyay , et al., “Amplification of the CXCR3/CXCL9 Axis via Intratumoral Electroporation of Plasmid CXCL9 Synergizes With Plasmid IL‐12 Therapy to Elicit Robust Anti‐Tumor Immunity,” Molecular Therapy Oncolytics 25 (2022): 174–188. 35592387 10.1016/j.omto.2022.04.005 PMC9092072

[45] P. Ma , J. Liu , J. Qin , et al., “Expansion of Pathogenic Cardiac Macrophages in Immune Checkpoint Inhibitor Myocarditis,” Circulation 149, no. 1 (2024): 48–66. 37746718 10.1161/CIRCULATIONAHA.122.062551 PMC11323830

[46] A. Pant , A. Jain , Y. Chen , et al., “The CCR6‐CCL20 Axis Promotes Regulatory T‐Cell Glycolysis and Immunosuppression in Tumors,” Cancer Immunology Research 12, no. 11 (2024): 1542–1558. 39133127 10.1158/2326-6066.CIR-24-0230

[47] S. L. Wang and T. A. Chan , “Navigating Established and Emerging Biomarkers for Immune Checkpoint Inhibitor Therapy,” Cancer Cell 43, no. 4 (2025): 641–664. 40154483 10.1016/j.ccell.2025.03.006 PMC13171157

[48] B. Song , K. Wang , S. Na , et al., “Profiling Antigen‐Binding Affinity of B Cell Repertoires in Tumors by Deep Learning Predicts Immune‐Checkpoint Inhibitor Treatment Outcomes,” Nature Cancer 6, no. 9 (2025): 1570–1584. 40579590 10.1038/s43018-025-01001-5 PMC13076093

[49] K. R. Monson , R. Ferguson , J. E. Handzlik , et al., “Inherited Mitochondrial Genetics as a Predictor of Immune Checkpoint Inhibition Efficacy in Melanoma,” Nature Medicine 31, no. 7 (2025): 2385–2396. 10.1038/s41591-025-03699-3 PMC12283385 40473950

[50] S. K. Yoo , C. W. Fitzgerald , B. A. Cho , et al., “Prediction of Checkpoint Inhibitor Immunotherapy Efficacy for Cancer Using Routine Blood Tests and Clinical Data,” Nature Medicine 31, no. 3 (2025): 869–880. 10.1038/s41591-024-03398-5 PMC11922749 39762425

[51] N. G. Nunez , F. Berner , E. Friebel , et al., “Immune Signatures Predict Development of Autoimmune Toxicity in Patients With Cancer Treated With Immune Checkpoint Inhibitors,” Med 4, no. 2 (2023): 113–129.e7. 36693381 10.1016/j.medj.2022.12.007

[52] G. D. Kim , S. I. Shin , P. Sun , et al., “Single‐Cell RNA Sequencing of Baseline PBMCs Predicts ICI Efficacy and irAE Severity in Patients With NSCLC,” Journal for ImmunoTherapy of Cancer 13, no. 5 (2025): e011636. 40404203 10.1136/jitc-2025-011636 PMC12097017

[53] X. Han , Y. Chen , H. Xie , et al., “Organ‐specific Immune‐Related Adverse Events and Prognosis in Cancer Patients Receiving Immune Checkpoint Inhibitors,” BMC Cancer 25, no. 1 (2025): 139. 39856626 10.1186/s12885-025-13566-6 PMC11761211

[54] S. M. Durbin , L. Zubiri , K. Perlman , et al., “Late‐Onset Immune‐Related Adverse Events After Immune Checkpoint Inhibitor Therapy,” JAMA Network Open 8, no. 3 (2025): e252668. 40146104 10.1001/jamanetworkopen.2025.2668 PMC11950896

[55] G. Kichenadasse , J. O. Miners , A. A. Mangoni , A. Rowland , A. M. Hopkins , and M. J. Sorich , “Multiorgan Immune‐Related Adverse Events During Treatment With Atezolizumab,” Journal of the National Comprehensive Cancer Network: JNCCN 18, no. 9 (2020): 1191–1199. 32886899 10.6004/jnccn.2020.7567

[56] G. Han , M. H. Spitzer , S. C. Bendall , W. J. Fantl , and G. P. Nolan , “Metal‐Isotope‐Tagged Monoclonal Antibodies for High‐Dimensional Mass Cytometry,” Nature Protocols 13, no. 10 (2018): 2121–2148. 30258176 10.1038/s41596-018-0016-7 PMC7075473

[57] E. R. Zunder , R. Finck , G. K. Behbehani , et al., “Palladium‐Based Mass Tag Cell Barcoding With a Doublet‐Filtering Scheme and Single‐Cell Deconvolution Algorithm,” Nature Protocols 10, no. 2 (2015): 316–333. 25612231 10.1038/nprot.2015.020 PMC4347881

[58] R. Finck , E. F. Simonds , A. Jager , et al., “Normalization of Mass Cytometry Data With Bead Standards,” Cytometry Part A: The Journal of the International Society for Analytical Cytology 83, no. 5 (2013): 483–494. 23512433 10.1002/cyto.a.22271 PMC3688049

[59] C. B. Pedersen , S. H. Dam , M. B. Barnkob , et al., “cyCombine Allows for Robust Integration of Single‐Cell Cytometry Datasets Within and Across Technologies,” Nature Communications 13, no. 1 (2022): 1698. 10.1038/s41467-022-29383-5 PMC8971492 35361793

[60] N. Samusik , Z. Good , M. H. Spitzer , K. L. Davis , and G. P. Nolan , “Automated Mapping of Phenotype Space with Single‐Cell Data,” Nature Methods 13, no. 6 (2016): 493–496. 27183440 10.1038/nmeth.3863 PMC4896314

[61] L. van der Maaten and G. Hinton , “Visualizing Data Using T‐SNE,” Journal of Machine Learning Research 9 (2008): 2579–2605.

[62] A. Behdenna , M. Colange , J. Haziza , et al., “pyComBat, a Python Tool for Batch Effects Correction in High‐Throughput Molecular Data Using Empirical Bayes Methods,” BMC Bioinformatics [Electronic Resource] 24, no. 1 (2023): 459. 38057718 10.1186/s12859-023-05578-5 PMC10701943

