# Supplementary material for: Peripheral Blood Mononuclear Cells Profiling Revealed Biomarkers That Predict PD‐1 Inhibitor‐Induced Immune‐Related Adverse Events
Source: MedComm (2020). 2026 Apr 5;7(4):e70721. doi: 10.1002/mco2.70721 (PMC13052260; doi:10.1002/mco2.70721)
Supplement: Supplementary file 1 — Supporting Figure 1: Protein markers expression levels between irAE and non_irAE group. Supporting Figure 2: TSNE plot illustrating the differential levels and distributions of 41 markers respectively. Supporting Figure 3: The frequencies of CyTOF events for each clusters between irAE and non_irAE group. Supporting Figure 4: CXCR3 and CCR6 expression levels in PBMC subpopulations between irAE and non_irAE group and their expression in corresponding PBMC subpopulations between PR and SD/PD group. Supporting Figure 5: PD‐1 and TIGIT expressions in PBMC subpopulations between irAE and non_irAE groups. Supporting Figure 6: Fas and CD25 expressions in PBMC subpopulations between irAE and non_irAE groups. Supporting Figure 7: Gating of Cluster 22. Supporting Figure 8: Full image of lung biopsy and partial image of lung gross examination results at same magnification. Supporting Figure 9: Full image of colon biopsy and partial image of colon cancer specimen results at same magnification. Supporting Figure 10: CCR6 expression in PBMC subpopulations of Cohort 3. Supporting Figure 11: Recapitulating clusters for Cohort 4 base on Cohort 1. Supporting Figure 12: Identification of differentially expressed protein markers between PR and SD/PD group. Supporting Figure 13: Identification of differentially expressed protein markers between PR and SD/PD group in PBMC subpopulations. Supporting Table 1: Detailed patient information of cohort 1 and cohort 2. Supporting Table 1 (continued): Detailed patient information of cohort 3 and cohort 4. Supporting Table 2: Iron channels and antibodies for CyTOF. [file MCO2-7-e70721-s001.pdf]

Supplementary Information to the manuscript entitled ‘Peripheral blood mononuclear cells profiling revealed biomarkers that predict PD-1 inhibitor-induced immune-related adverse events’

By Jun Wang et al.,

Submitted to *MedComm*

Content

Page 2, Figure S1
Page 4, Figure S2
Page 5, Figure S3
Page 6, Figure S4
Page 8, Figure S5
Page 9, Figure S6
Page 10, Figure S7
Page 11, Figure S8
Page 12, Figure S9
Page 13, Figure S10
Page 14, Figure S11
Page 16, Figure S12
Page 17, Figure S13
Page 18, Table S 1
Page 19, Table S1 (continued)
Page 20, Table S2

Figure S1. Protein markers expression levels between irAE and non_irAE group

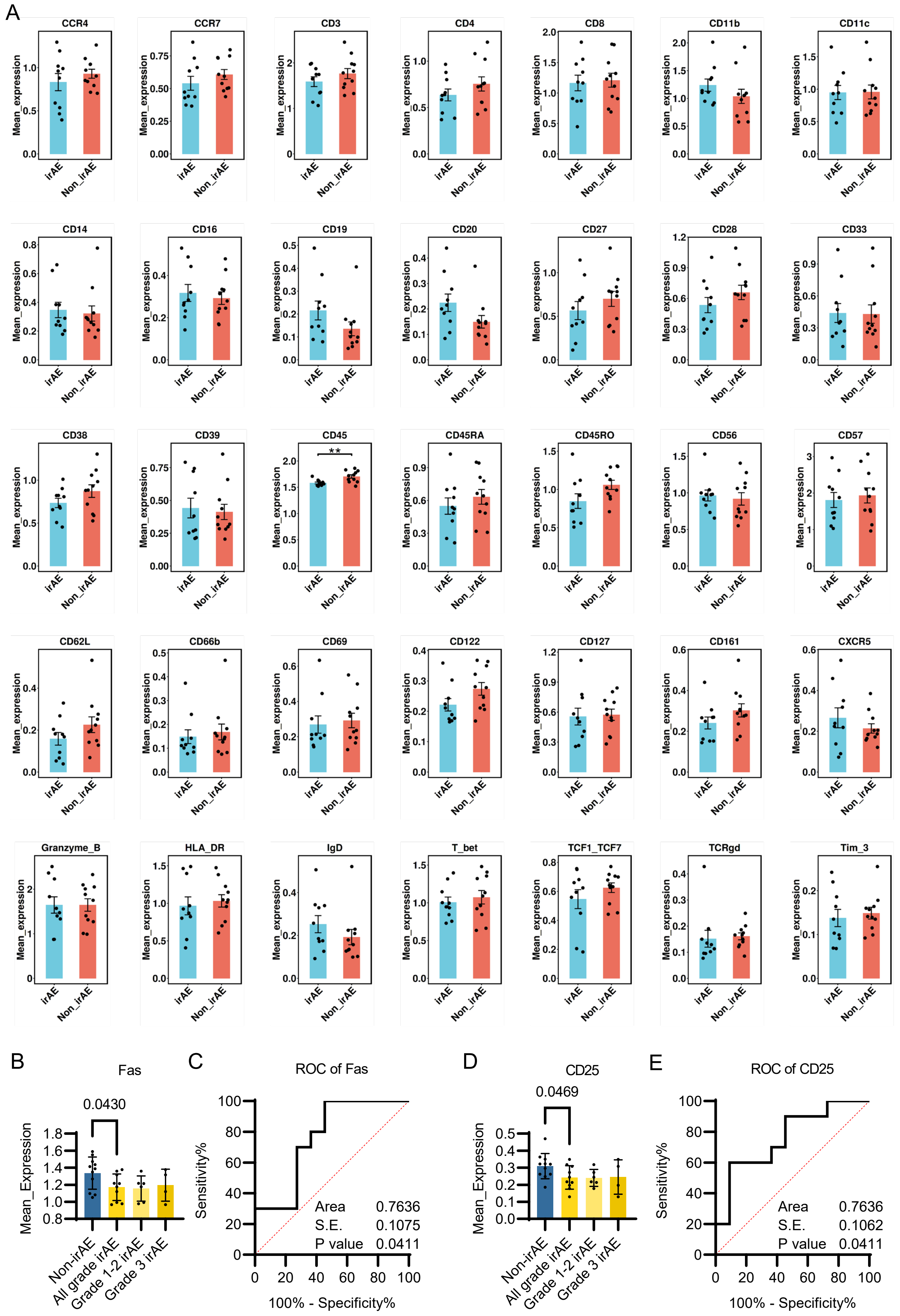

Figure S1. Protein markers expression levels between irAE and non_irAE group

A. Protein markers expression levels between irAE and non_irAE group
B. Fas expression levels in non_irAE group, irAE group, grade 1-1 irAE group and grade 3 irAE group
C. ROC curve and AUC of Fas (AUC: 0.7636, 95% CI: 0.55294 to 0.9743, *p* value: 0.0411) in predicting occurrence of all grade irAEs
D. CD25 expression levels in non_irAE group, irAE group, grade 1-1 irAE group and grade 3 irAE group
E. ROC curve and AUC of CD25 (AUC: 0.7636, 95% CI: 0.5555 to 0.9718, *p* value: 0.0411) in predicting occurrence of all grade irAEs

Figure S2. TSNE plot illustrating the differential levels and distributions of 41 markers respectively

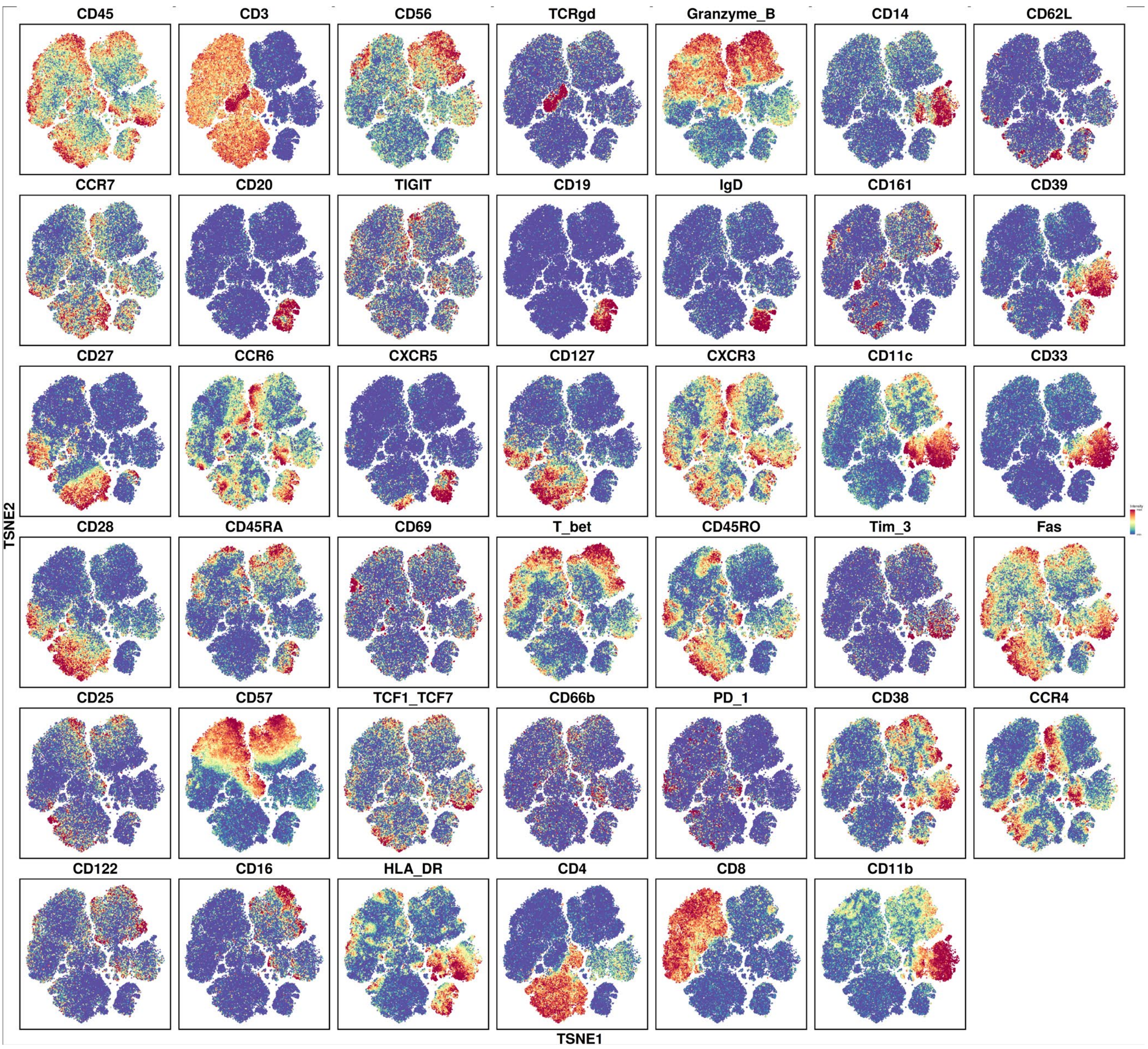

Distributions and levels of 41 markers respectively visualized by TSNE plot

Figure S3. The frequencies of CyTOF events for each clusters between irAE and non_irAE group

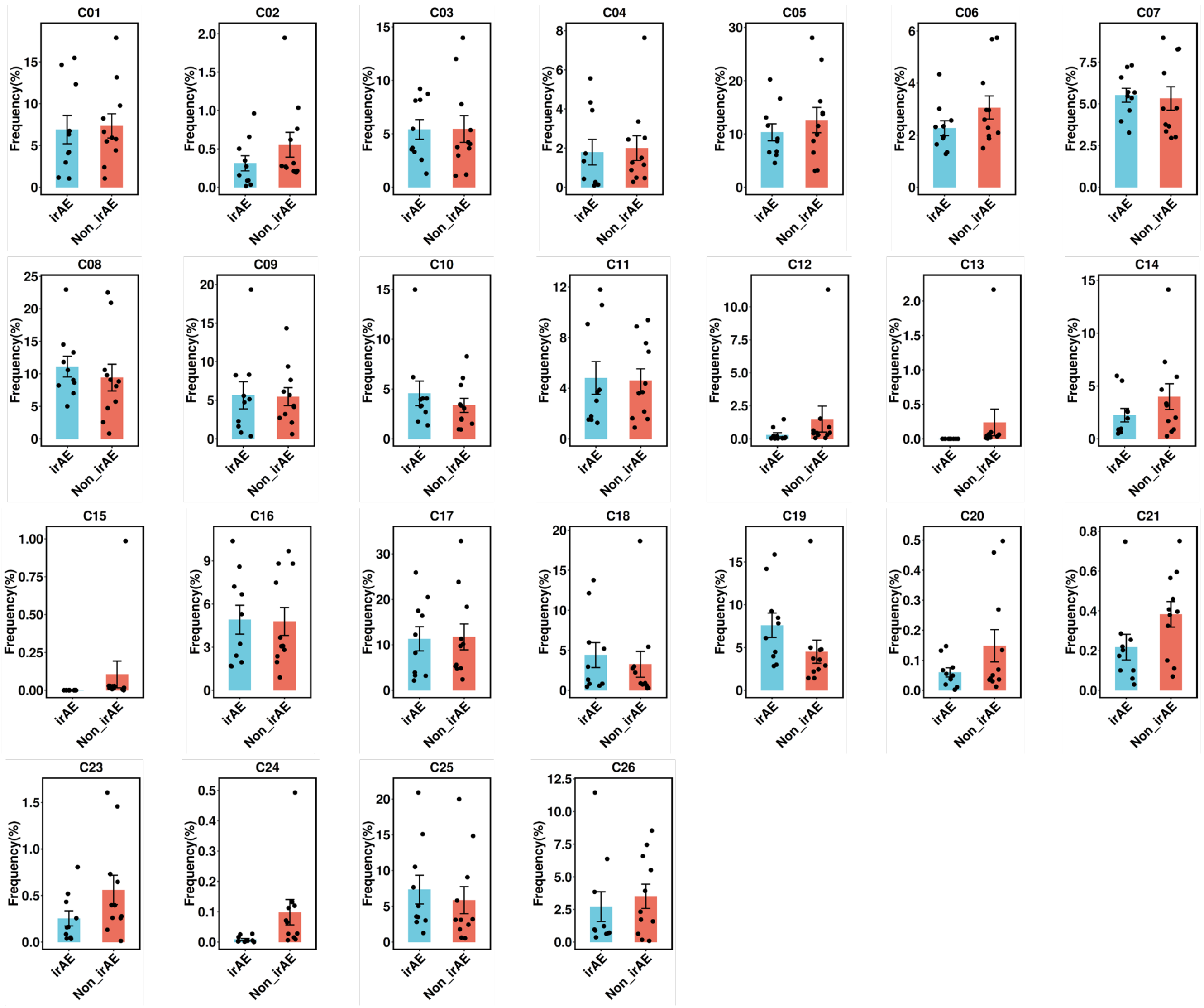

The frequencies of each clusters identified by the CyTOF between irAE and non_irAE group

Figure S4. CXCR3 and CCR6 expression levels in PBMC subpopulations between irAE and non_irAE group and their expression in corresponding PBMC subpopulations between PR and SD/PD group

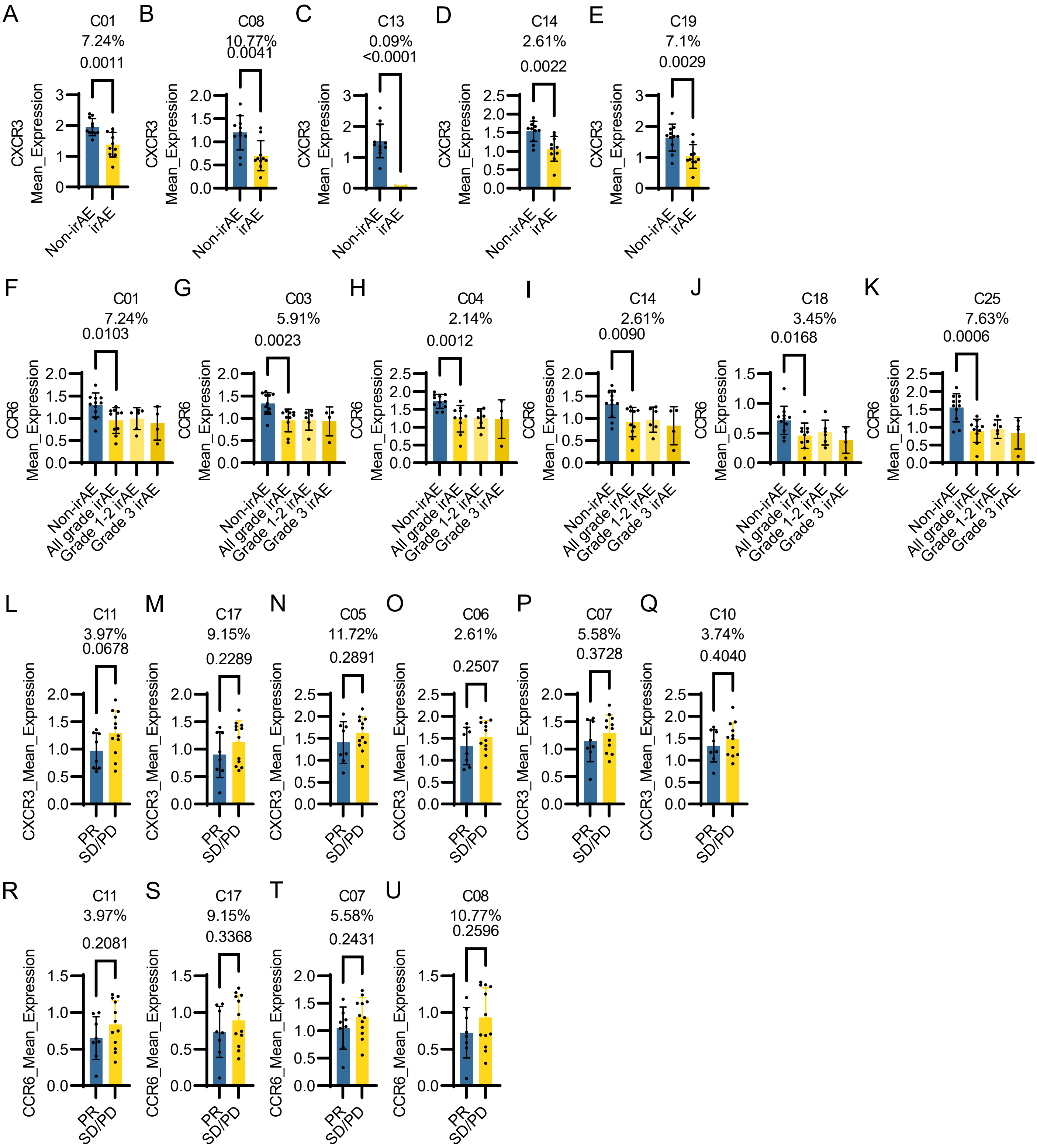

Figure S4. CXCR3 and CCR6 expression levels in PBMC subpopulations between irAE and non_irAE group and their expression in corresponding PBMC subpopulations between PR and SD/PD group

A. Expression levels of CXCR3 in cluster 01 between irAE and non_irAE groups
B. Expression levels of CXCR3 in cluster 08 between irAE and non_irAE groups
C. Expression levels of CXCR3 in cluster 13 between irAE and non_irAE groups
D. Expression levels of CXCR3 in cluster 14 between irAE and non_irAE groups
E. Expression levels of CXCR3 in cluster 19 between irAE and non_irAE groups
F. Expression levels of CCR6 in cluster 01 among non_irAE, all grades irAE, grade 1-2 irAE and grade 3 groups
G. Expression levels of CCR6 in cluster 03 among non_irAE, all grades irAE, grade 1-2 irAE and grade 3 groups
H. Expression levels of CCR6 in cluster 04 among non_irAE, all grades irAE, grade 1-2 irAE and grade 3 groups
I. Expression levels of CCR6 in cluster 14 among non_irAE, all grades irAE, grade 1-2 irAE and grade 3 groups
J. Expression levels of CCR6 in cluster 18 among non_irAE, all grades irAE, grade 1-2 irAE and grade 3 groups
K. Expression levels of CCR6 in cluster 25 among non_irAE, all grades irAE, grade 1-2 irAE and grade 3 groups
L. Expression levels of CXCR3 in cluster 11 between PR and SD/PD groups
M. Expression levels of CXCR3 in cluster 17 between PR and SD/PD groups
N. Expression levels of CXCR3 in cluster 05 between PR and SD/PD groups
O. Expression levels of CXCR3 in cluster 06 between PR and SD/PD groups
P. Expression levels of CXCR3 in cluster 07 between PR and SD/PD groups
Q. Expression levels of CXCR3 in cluster 10 between PR and SD/PD groups
R. Expression levels of CCR6 in cluster 11 between PR and SD/PD groups
S. Expression levels of CCR6 in cluster 17 between PR and SD/PD groups
T. Expression levels of CCR6 in cluster 07 between PR and SD/PD groups
U. Expression levels of CCR6 in cluster 08 between PR and SD/PD groups

Figure S5. PD-1 and TIGIT expressions in PBMC subpopulations between irAE and non_irAE groups

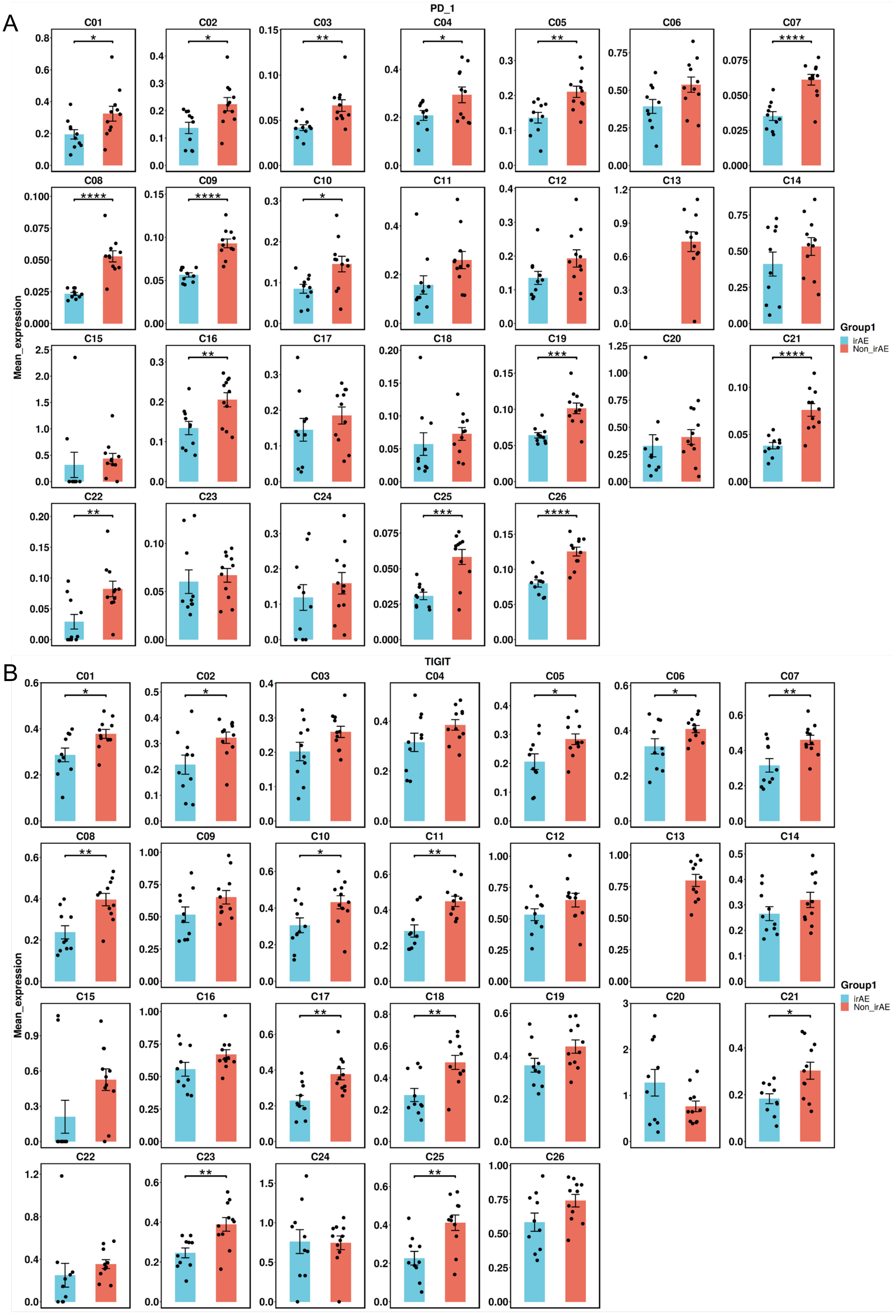

A. PD-1 expressions in PBMC subpopulations between irAE and non_irAE groups
B. TIGIT expressions in PBMC subpopulations between irAE and non_irAE groups

Figure S6. Fas and CD25 expressions in PBMC subpopulations between irAE and non_irAE groups

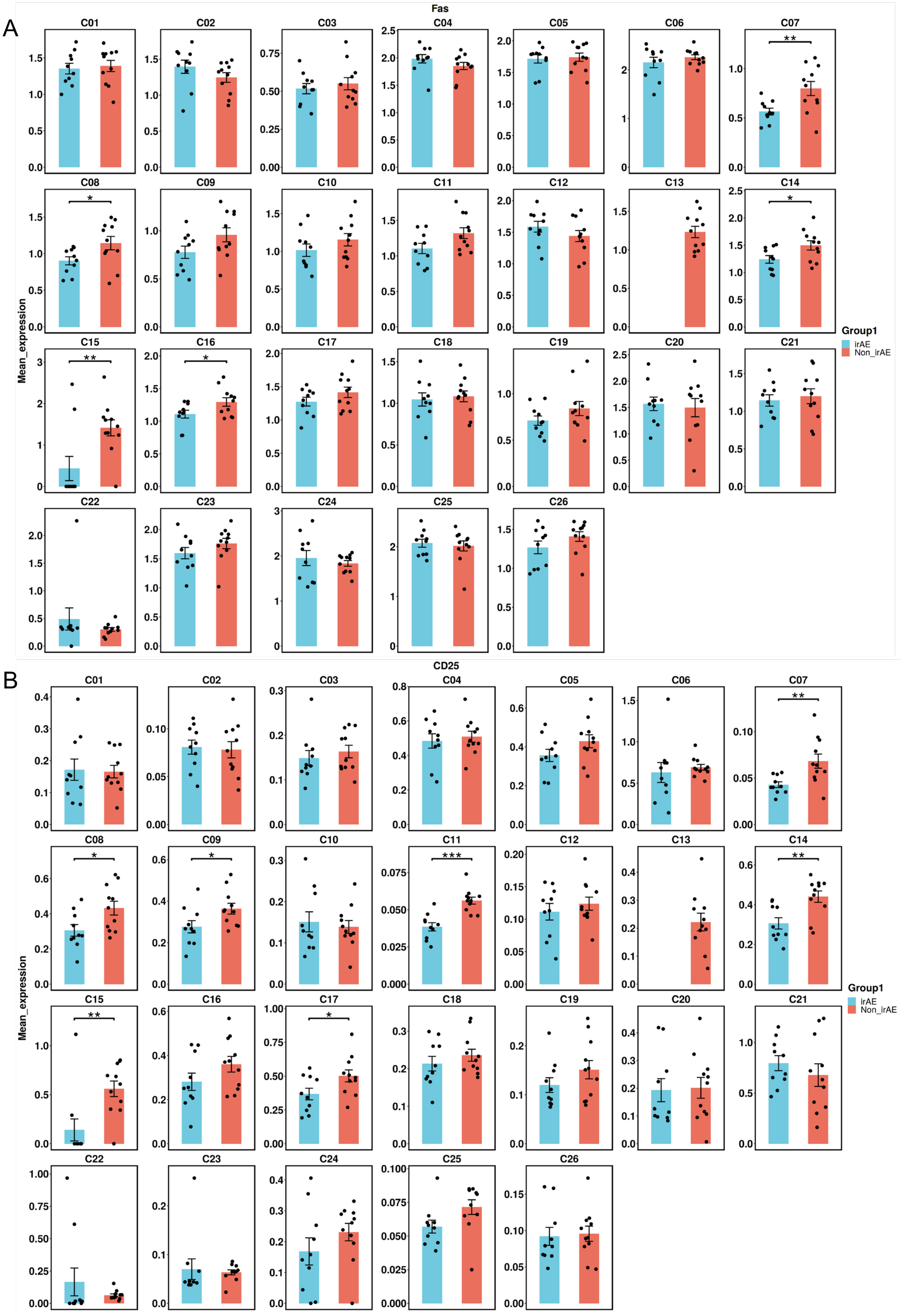

A. Fas expressions in PBMC subpopulations between irAE and non_irAE groups
B. CD25 expressions in PBMC subpopulations between irAE and non_irAE groups

Figure S7. Gating of Cluster 22

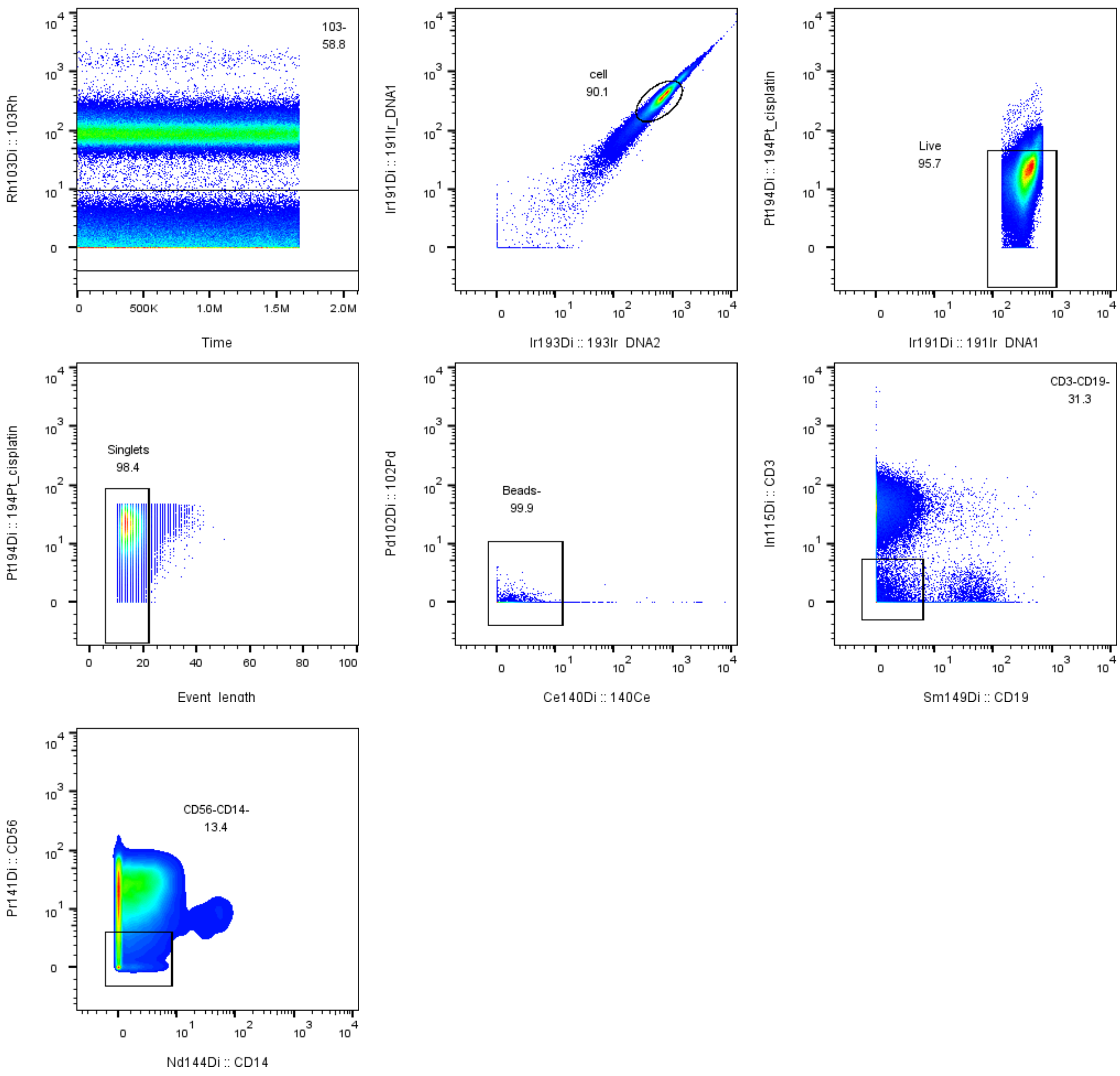

Gating method to identify Cluster 22

Figure S8. Full image of lung biopsy and partial image of lung gross examination results at same magnification

A

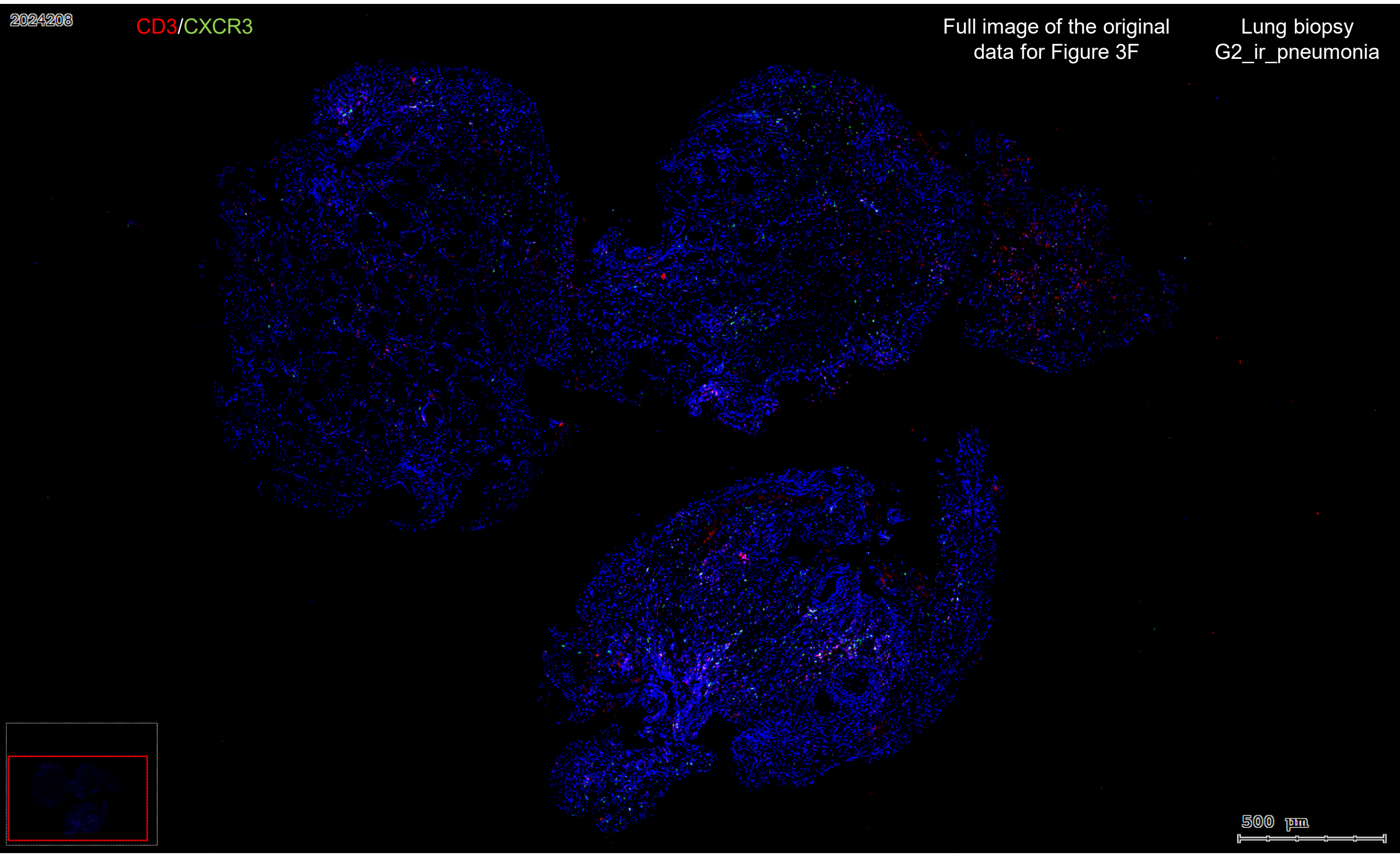

B

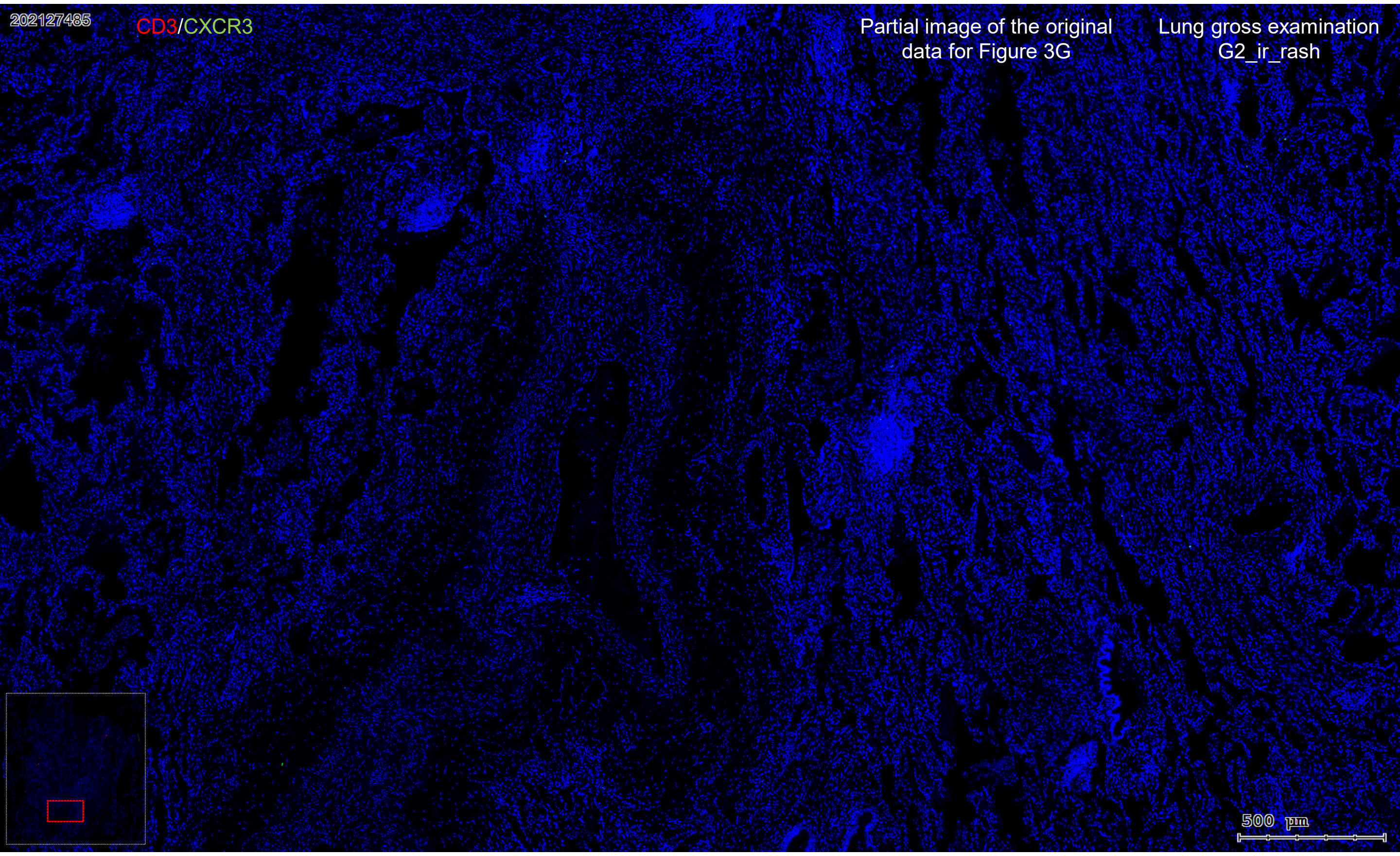

A. Full image of the original data for Figure 3F
B. Partial image of the original data for Figure 3G

Figure S9. Full image of colon biopsy and partial image of colon cancer specimen results at same magnification

A

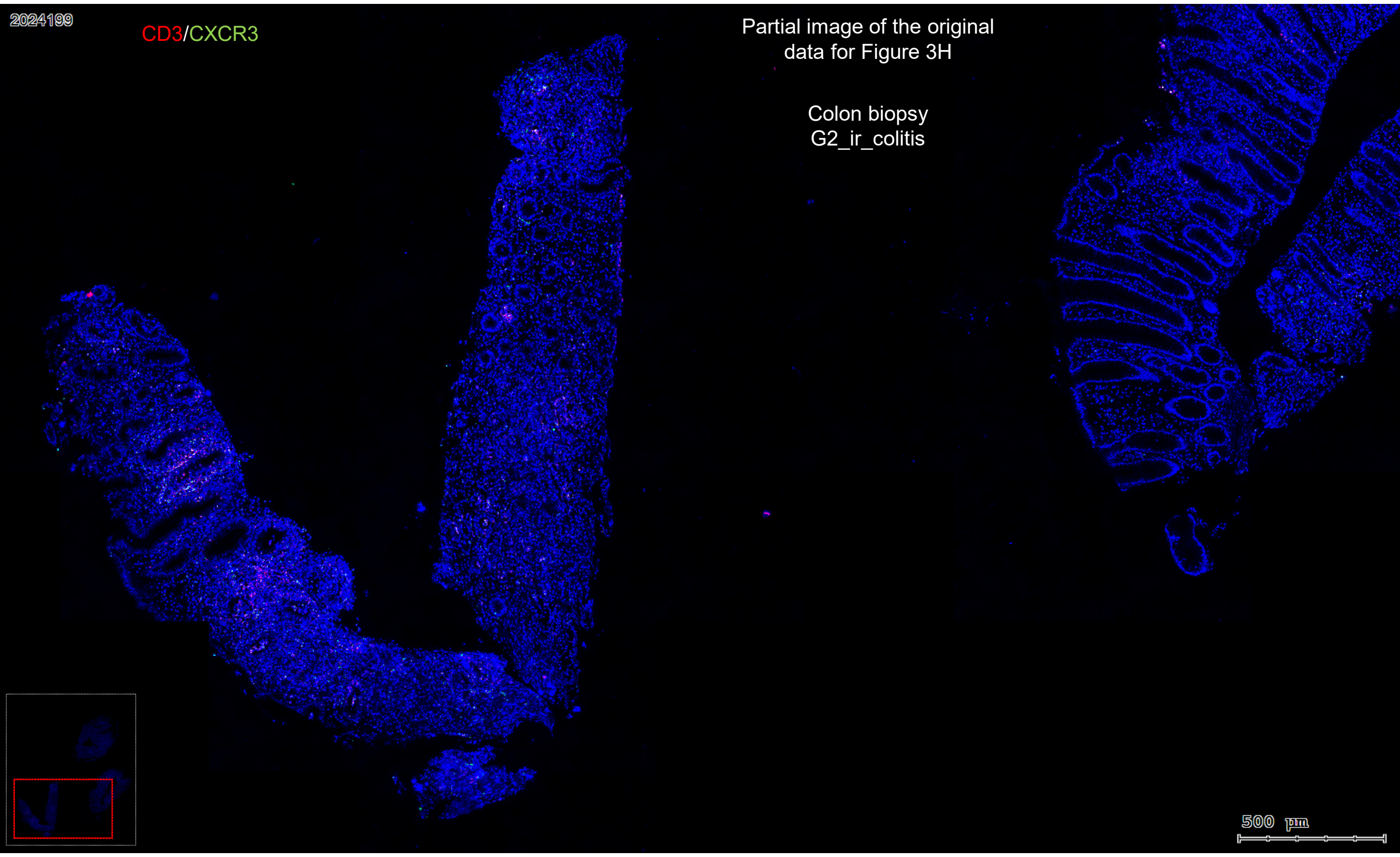

B

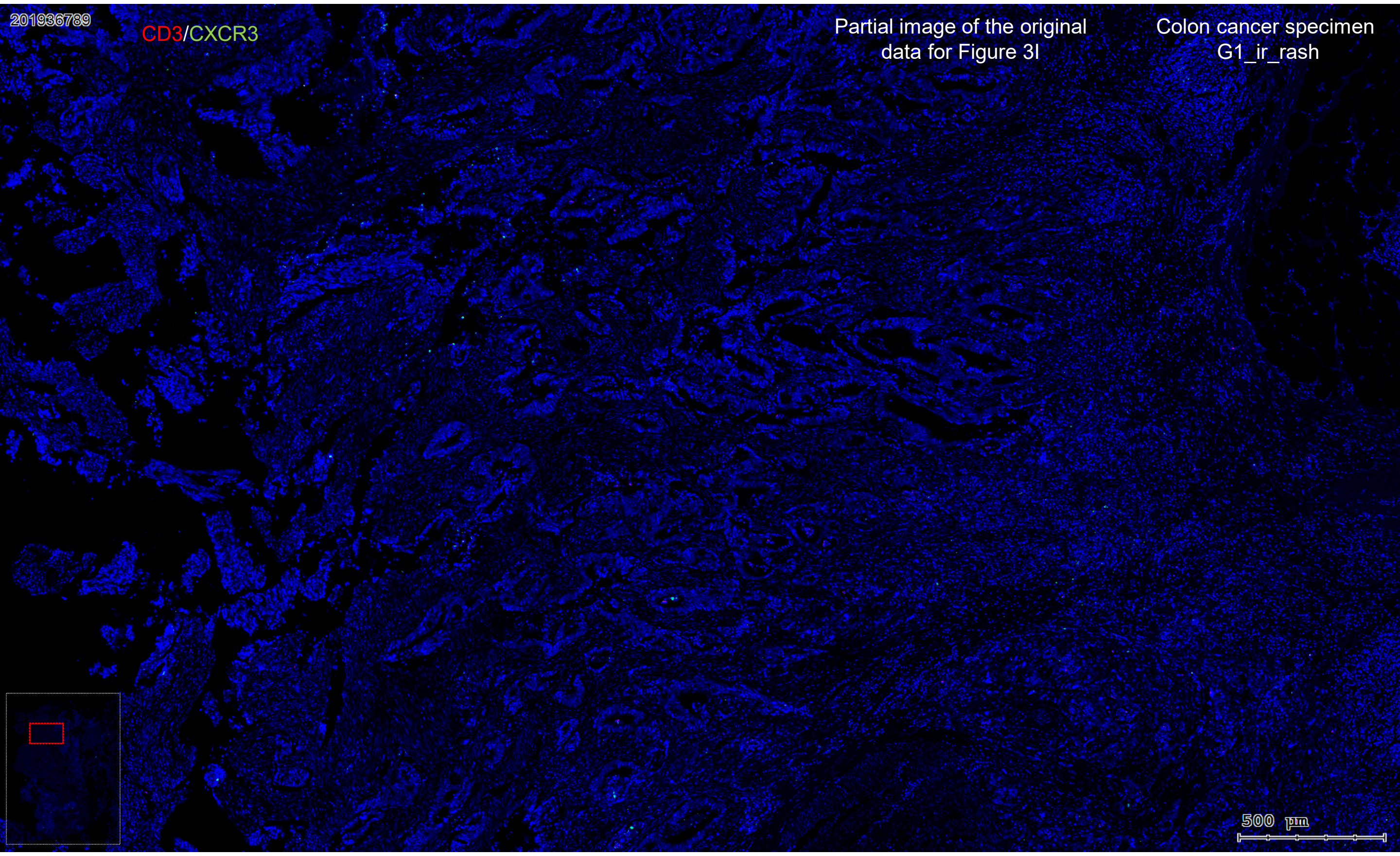

A. Full image of the original data for Figure 3H
B. Partial image of the original data for Figure 3I

Figure S10. CCR6 expression in PBMC subpopulations of Cohort 3

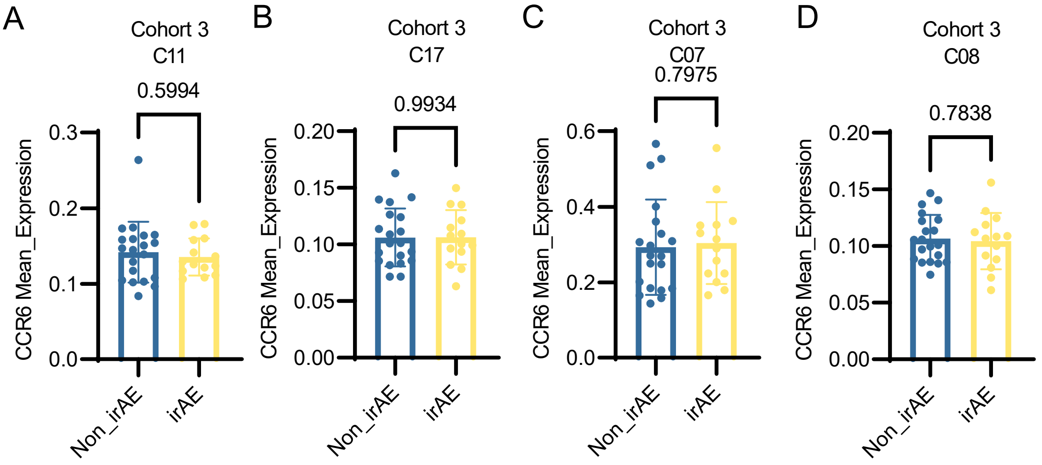

A. Expression levels of CCR6 in cluster 11 of validation Cohort 3 between irAE and non_irAE groups
B. Expression levels of CCR6 in cluster 17 of validation Cohort 3 between irAE and non_irAE groups
C. Expression levels of CCR6 in cluster 07 of validation Cohort 3 between irAE and non_irAE groups
D. Expression levels of CCR6 in cluster 08 of validation Cohort 3 between irAE and non_irAE groups

Figure S11. Recapitulating clusters for Cohort 4 base on Cohort 1

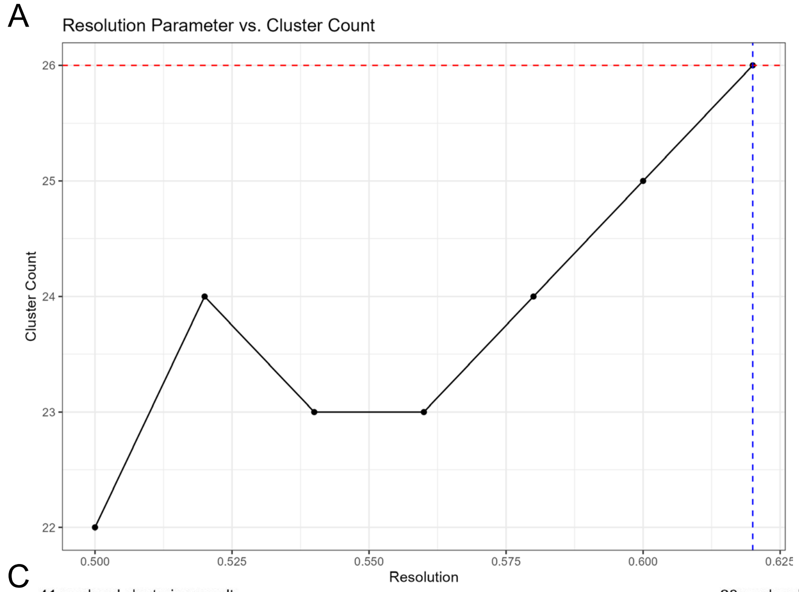

B

Cohort1_cluster	Cohort4_cluster	similarity
C17	cluster_0	0.94935
C03	cluster_1	0.953953
C16	cluster_10	0.966886
C25	cluster_11	0.98228
C11	cluster_12	0.905893
C14	cluster_13	0.967356
C06	cluster_16	0.924441
C12	cluster_17	0.956919
C20	cluster_19	0.898971
C05	cluster_2	0.963765
C22	cluster_21	0.93526
C21	cluster_22	0.96546
C24	cluster_23	0.918439
C01	cluster_3	0.92139
C09	cluster_4	0.975551
C08	cluster_5	0.973771
C19	cluster_7	0.969993
C26	cluster_8	0.978341
C07	cluster_9	0.938331

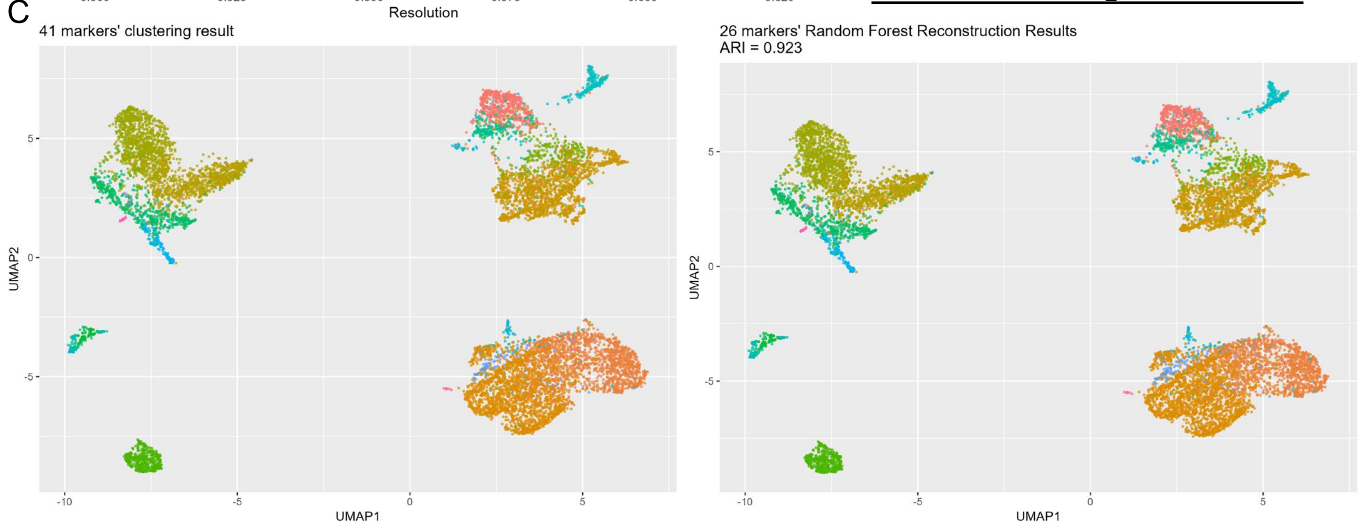

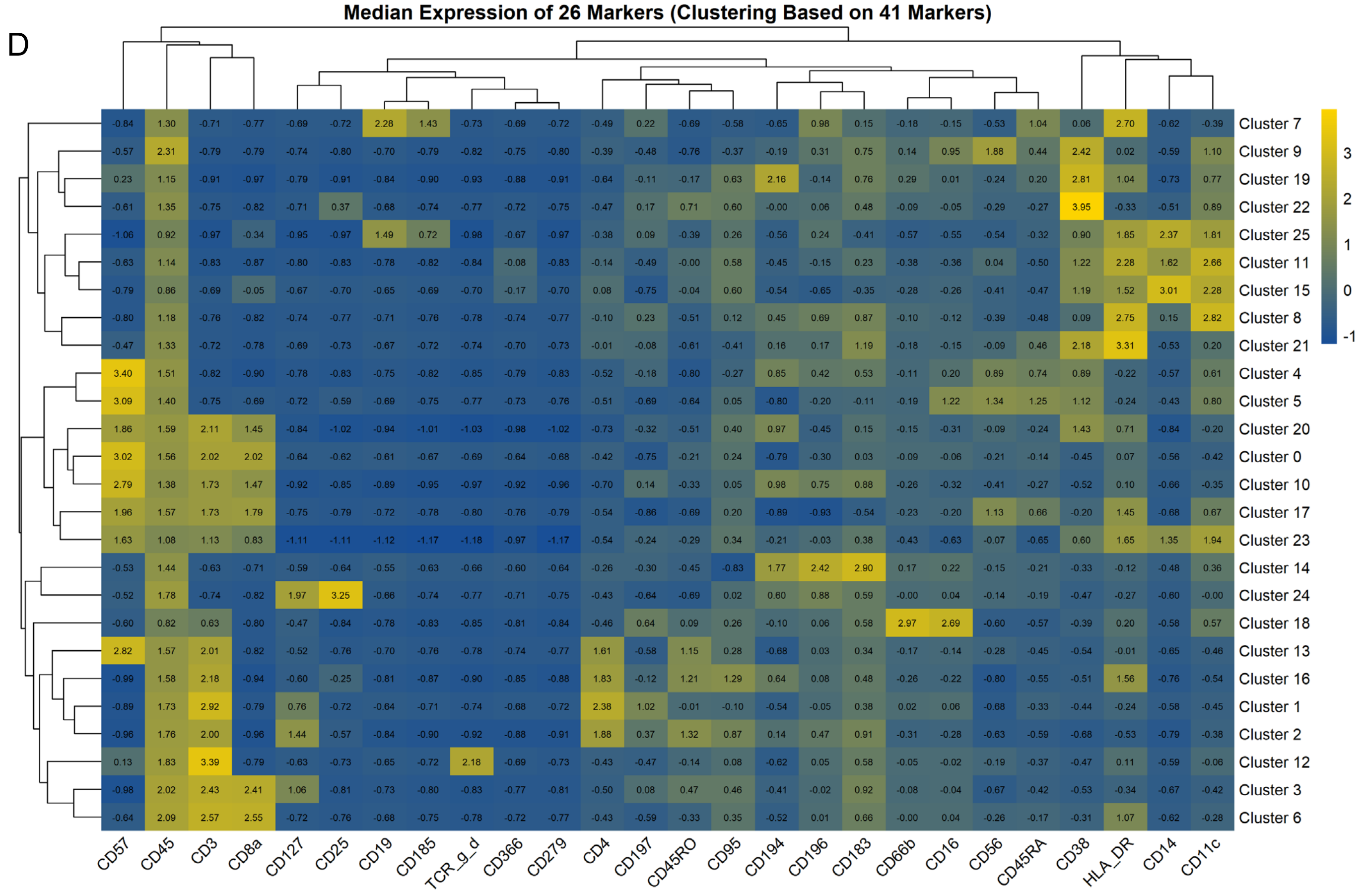

Figure S11. Recapitulating clusters for Cohort 4 base on Cohort 1

A. Cluster count adjustment to reach the fit resolution

B. Similarity between clusters from Cohort 1 and Cohort 4

C. Recapitulated clusters for Cohort 4 base on Cohort 1 with an ARI of 0.923

D. Median expression of 26 makers of Cohort 4 in the recapitulated clusters

Figure S12. Identification of differentially expressed protein markers between PR and SD/PD group

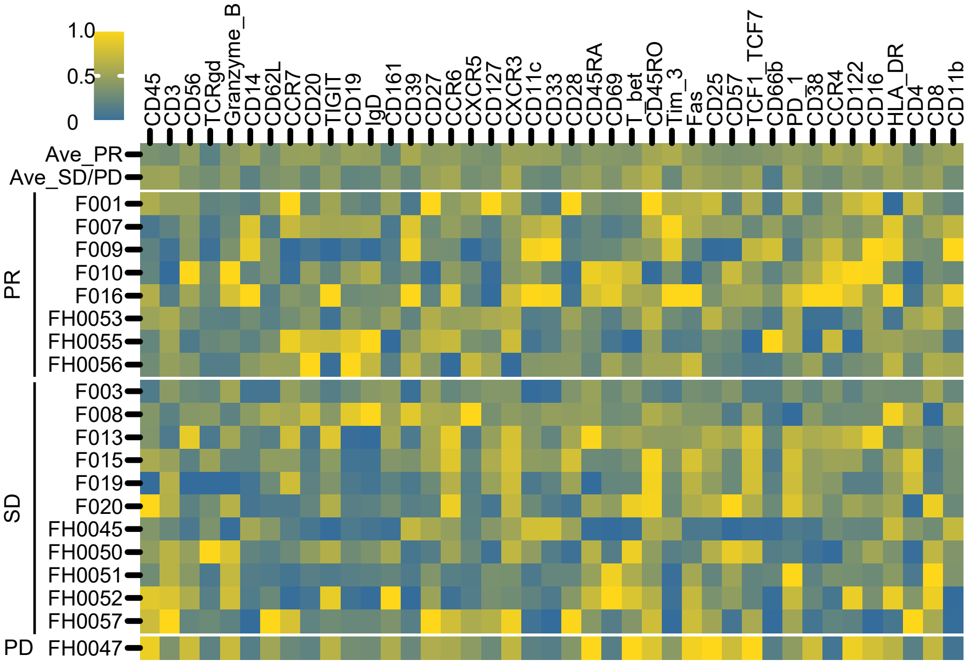

Full heatmap showing differentially expressed protein markers of each patient between PR and SD/PD group

Figure S13. Identification of differentially expressed protein markers between PR and SD/PD group in PBMC subpopulations

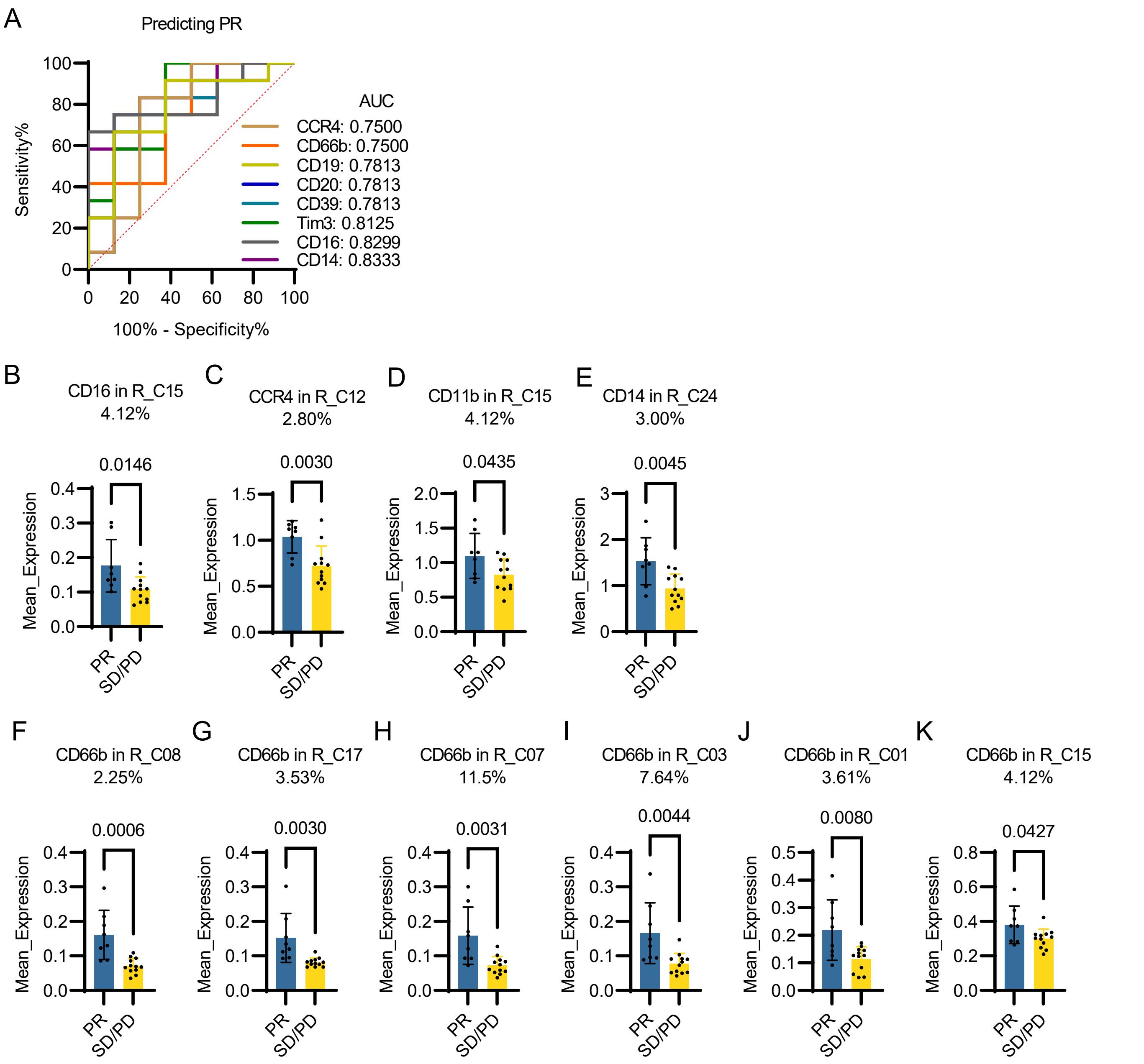

Figure S13. Identification of differentially expressed protein markers between PR and SD/PD group in PBMC subpopulations

A. ROC curves and AUCs of CCR4, CD66b, CD19, CD20, CD39, Tim3, CD16 and CD14 in predicting response to PD-1 treatment
B. Expression levels of CD16 in R_C15 between PR and SD/PD groups
C. Expression levels of CCR4 in R_C12 between PR and SD/PD groups
D. Expression levels of CD11b in R_C15 between PR and SD/PD groups
E. Expression levels of CD14 in R_C24 between PR and SD/PD groups
F. Expression levels of CD66b in R_C08 between PR and SD/PD groups
G. Expression levels of CD66b in R_C17 between PR and SD/PD groups
H. Expression levels of CD66b in R_C07 between PR and SD/PD groups
I. Expression levels of CD66b in R_C03 between PR and SD/PD groups
J. Expression levels of CD66b in R_C01 between PR and SD/PD groups
K. Expression levels of CD66b in R_C15 between PR and SD/PD groups

Table S1. Detailed patient information of cohort 1 and cohort 2

Supplementary Table 1

Cohort 1

Sample ID	irAE Status	Time of irAE onset(d)	irAE type	irAE grade	Response	Diagnosis	ICI	PD-1/PD-L1	Combination regimen	ECOG	ICI cycles	Multiple irAEs
F001	irAE	48	Cutaneous	3	PR	Lung adenocarcinoma	Camrelizumab	PD-1	Abraxane+Carboplatin	1	4	No
F003	irAE	98	Cutaneous	2	SD	Lung squamous carcinoma	Pembrolizumab	PD-1	Abraxane+Carboplatin	1	4	No
F007	irAE	171	Cutaneous	1	PR	Lung squamous carcinoma	Sintilimab	PD-1	Paclitaxel Liposome+Carboplatin	0	5	No
F008	irAE	45	Pneumonic	2	SD	Lung adenocarcinoma	Camrelizumab	PD-1	ALIMTA+Carboplatin	1	3	No
F009	irAE	101	Enteritic	3	PR	Kidney renal clear cell carcinoma	Sintilimab	PD-1	Sunitinib Malate	1	3	Grade 2 Enteritis; Grade 1 Retinitis
F010	irAE	71	Pneumonic	2	PR	Lung squamous carcinoma	Camrelizumab	PD-1	Abraxane+Carboplatin	1	3	No
F013	Non_irAE	N.A.	N.A.	0	SD	Large-cell neuroendocrine carcinoma of the lung	Sintilimab	PD-1	Abraxane	2	2	
F015	Non_irAE	N.A.	N.A.	0	SD	Kidney renal clear cell carcinoma	Tislelizumab	PD-1	Sunitinib	0	3	
F016	Non_irAE	N.A.	N.A.	0	PR	Lung adenocarcinoma	Sintilimab	PD-1	Etoposide+Cisplatin	1	15	
F019	Non_irAE	N.A.	N.A.	0	SD	Stomach adenocarcinoma	Sintilimab	PD-1	XELOX	2	6	
F020	Non_irAE	N.A.	N.A.	0	SD	Cholangio carcinoma	Sintilimab	PD-1	Gemcitabine + Cisplatin	0	7	
FH0045	Non_irAE	N.A.	N.A.	0	SD	Stomach adenocarcinoma	Sintilimab	PD-1	XELOX	1	6	
FH0047	Non_irAE	N.A.	N.A.	0	PD	Lung adenocarcinoma	Camrelizumab	PD-1	ALIMTA+Carboplatin	0	2	
FH0050	irAE	178	Endocrinic	2	SD	Melanoma	Toripalimab	PD-1	No	0	6	Grade 2 Hypothyroidism; Grade 1 Myositis, Hepatitis
FH0051	irAE	145	Pneumonic	2	SD	Lung adenocarcinoma	Camrelizumab	PD-1	ALIMTA+Carboplatin	1	6	No
FH0052	Non_irAE	N.A.	N.A.	0	SD	Stomach adenocarcinoma	Sintilimab	PD-1	XELOX	0	2	
FH0053	irAE	498	Cutaneous	3	PR	Lung adenocarcinoma	Tislelizumab	PD-1	ALIMTA+Carboplatin	1	24	No
FH0054	Non_irAE	N.A.	N.A.	0	SD	Stomach adenocarcinoma	Sintilimab	PD-1	Abraxane	1	2	
FH0055	Non_irAE	N.A.	N.A.	0	PR	Stomach adenocarcinoma	Sintilimab	PD-1	Oxa+LV+5-FU	2	10	
FH0056	irAE	520	Pneumonic	3	PR	Stomach adenocarcinoma	Sintilimab	PD-1	Oxa+LV+5-FU	0	8	No
FH0057	Non_irAE	N.A.	N.A.	0	SD	Stomach adenocarcinoma	Sintilimab	PD-1	Abraxane+Oxa+5-FU	1	2	
F011	Non_irAE	N.A.	N.A.	0	SD	Lung squamous carcinoma	Camrelizumab	PD-1	Nab-paclitaxel+nedaplatin	1	6	
F017	Non_irAE	N.A.	N.A.	0	PR	Stomach adenocarcinoma	Sintilimab	PD-1	XELOX	1	2	
F015	Non_irAE	N.A.	N.A.	0	PD	Small cell lung cancer	Sintilimab	PD-1	Etoposide+Carboplatin	1	3	
F018	Non_irAE	N.A.	N.A.	0	SD	Squamous cell carcinoma of skin	Camrelizumab	PD-1	Gemcitabine + Cisplatin	2	2	
FH0070	Non_irAE	N.A.	N.A.	0	SD	Stomach adenocarcinoma	Sintilimab	PD-1	Nab-paclitaxel	1	2	
FH0071	Non_irAE	N.A.	N.A.	0	PR	Lung adenocarcinoma	Sintilimab	PD-1	ALIMTA+Carboplatin	1	12	
F004	irAE	86	Pneumonic	4	SD	Stomach adenocarcinoma	Sintilimab	PD-1	XELOX	1	4	No
F005	irAE	52	Cardiac	4	SD	Colon adenocarcinoma	Sintilimab	PD-1	XELOX	1	2	Grade 4 Myocarditis; Grade 1 Hepatitis
F006	irAE	171	Pneumonic	3	PD	Kidney renal clear cell carcinoma	Sintilimab	PD-1	Paclitaxel Liposome+Carboplatin	1	2	No
FH0072	irAE	41	Pneumonic	2	SD	Stomach adenocarcinoma	Camrelizumab	PD-1	ALIMTA+Carboplatin	1	3	No
FH0073	irAE	493	Enteritic	3	PR	Kidney renal clear cell carcinoma	Sintilimab	PD-1	Sunitinib	1	3	No

Cohort 2

Sample ID	irAE Satus	Time of irAE onset(d)	irAE type	irAE grade	Response	Diagnosis	ICI	PD-1/PD-L1	Combination regimen	ECOG
202214443	irAE	20	Pneumonic	2	PR	Lung adenocarcinoma	Pembrolizumab	PD-1	No	1
202127485	irAE	28	Cutaneous	2	SD	Lung adenocarcinoma	Camrelizumab	PD-1	Amuvatinib	1
202430091	irAE	116	Enteritic	2	SD	Stomach adenocarcinoma	Sintilimab	PD-1	Capecitabine	1
201936789	irAE	240	Cutaneous	2	SD	Rectum adenocarcinoma	Camrelizumab	PD-1	Fruquintinib	1

Detailed patient information of cohort 1 and cohort 2 including Sample ID, irAE information, pathological type, ICI used and et al.

Table S1 (continued). Detailed patient information of cohort 3 and cohort 4

Supplementary Table 1 (continued)								
Cohort 3								
Patient ID	Age at surg	Pathological type		irAE grade	irAE type	NRS score	ICI	
FH0109	68	Esophageal Squamous	Non_irAE	0	None	1	Pembrolizumab	
FH0110	65	Esophageal Squamous	irAE	3	Endocrinic	1	Pembrolizumab	
FH0111	50	Esophageal Squamous	irAE	2	Cutaneous	4	Pembrolizumab	
FH0112	69	Esophageal Squamous	irAE	2	Cutaneous	4	Pembrolizumab	
FH0113	67	Esophageal Squamous	irAE	3	Enteritic	1	Pembrolizumab	
FH0114	66	Esophageal Squamous	irAE	1	Cutaneous	1	Pembrolizumab	
FH0115	67	Esophageal Squamous	irAE	2	Endocrinic	2	Pembrolizumab	
FH0116	52	Esophageal Squamous	Non_irAE	0	None	1	Pembrolizumab	
FH0117	57	Esophageal Squamous	Non_irAE	0	None	2	Pembrolizumab	
FH0118	66	Esophageal Squamous	Non_irAE	0	None	1	Pembrolizumab	
FH0134	65	Esophageal Squamous	Non_irAE	0	None	1	Pembrolizumab	
FH0136	53	Esophageal Squamous	Non_irAE	0	None	1	Pembrolizumab	
FH0138	69	Esophageal Squamous	Non_irAE	0	None	1	Pembrolizumab	
FH0139	77	Esophageal Squamous	Non_irAE	0	None	3	Pembrolizumab	
FH0140	58	Esophageal Squamous	Non_irAE	0	None	2	Pembrolizumab	
FH0141	70	Esophageal Squamous	Non_irAE	0	None	1	Pembrolizumab	
FH0142	67	Esophageal Squamous	irAE	1	Myonitis	1	Toripalimab	
FH0143	55	Esophageal Squamous	Non_irAE	0	None	1	Pembrolizumab	
FH0145	54	Esophageal Squamous	Non_irAE	0	None	1	Pembrolizumab	
FH0146	48	Esophageal Squamous	Non_irAE	0	None	1	Pembrolizumab	
FH0150	57	Esophageal Squamous	irAE	3	Endocrinic	4	Pembrolizumab	
FH0151	61	Esophageal Squamous	irAE	2	Cutaneous	2	Pembrolizumab	
FH0152	53	Esophageal Squamous	irAE	1	Cutaneous	1	Pembrolizumab	
FH0153	54	Esophageal Squamous	Non_irAE	0	None	1	Pembrolizumab	
FH0154	66	Esophageal Squamous	Non_irAE	0	None	1	Pembrolizumab	
FH0156	65	Esophageal Squamous	Non_irAE	0	None	1	Pembrolizumab	
FH0184	73	Esophageal Squamous	Non_irAE	0	None	2	Sintilimab	
FH0185	62	Esophageal Squamous	Non_irAE	0	None	1	Pembrolizumab	
FH0187	76	Esophageal Squamous	Non_irAE	0	None	2	Pembrolizumab	
FH0188	59	Esophageal Squamous	irAE	2	Cutaneous	1	Pembrolizumab	
FH0189	52	Esophageal Squamous	irAE	3	Hepatitic	1	Pembrolizumab	
FH0192	57	Esophageal Squamous	irAE	1	Myonitis	1	Pembrolizumab	
FH0193	65	Esophageal pleomorph	irAE	3	Endocrinic	1	Sintilimab	
FH0194	75	Esophageal Squamous	Non_irAE	0	None	2	Pembrolizumab	
Cohort 4								
Patient ID		Pathological type	irAE status	irAE grade		Time of irAE onset(d)	ICI	ICI cycles
Pre-001	78	Small Cell Lung Cancer	Non_irAE	0	None	N.A.	Durvalumab	6
Pre-002	50	Small Cell Lung Cancer	Non_irAE	0	None	N.A.	Atezolizumab	5
Pre-003	67	Small Cell Lung Cancer	Non_irAE	0	None	N.A.	Durvalumab	7
Pre-004	68	Small Cell Lung Cancer	irAE	2	Cutaneous	429	Durvalumab	14
Pre-005	61	Small Cell Lung Cancer	Non_irAE	0	None	N.A.	Atezolizumab	12
Pre-006	67	Small Cell Lung Cancer	Non_irAE	0	None	N.A.	Durvalumab	13
Pre-007	55	Small Cell Lung Cancer	Non_irAE	0	None	N.A.	Durvalumab	4
Pre-008	66	Small Cell Lung Cancer	Non_irAE	0	None	N.A.	Atezolizumab	9
Pre-009	35	Small Cell Lung Cancer	Non_irAE	0	None	N.A.	Atezolizumab	4
Pre-010	53	Small Cell Lung Cancer	Non_irAE	0	None	N.A.	Durvalumab	6
Pre-011	65	Small Cell Lung Cancer	Non_irAE	0	None	N.A.	Atezolizumab	4
Pre-012	54	Small Cell Lung Cancer	Non_irAE	0	None	N.A.	Atezolizumab	9
Pre-013	54	Small Cell Lung Cancer	Non_irAE	0	None	N.A.	Durvalumab	4
Pre-014	42	Small Cell Lung Cancer	Non_irAE	0	None	N.A.	Atezolizumab	6
Pre-015	66	Small Cell Lung Cancer	Non_irAE	0	None	N.A.	Durvalumab	10
Pre-016	82	Small Cell Lung Cancer	irAE	2	Enteritic	98	Durvalumab	4
Pre-017	59	Small Cell Lung Cancer	irAE	1	Pneumonic	223	Atezolizumab	10
Pre-018	60	Small Cell Lung Cancer	Non_irAE	0	None	N.A.	Durvalumab	8
Pre-019	60	Small Cell Lung Cancer	Non_irAE	0	None	N.A.	Atezolizumab	5
Pre-020	39	Small Cell Lung Cancer	Non_irAE	0	None	N.A.	Atezolizumab	8

Detailed patient information of cohort 1 and cohort 2 including Sample ID, irAE information, pathological type, ICI used and et al.

Table S2. Iron channels and antibodies for CyTOF

Supplementary Table 2. Iron channels and antibodies for Cytof						
Channel	Iron	Antibody	Antibody clone No.	Antibody Brand	Antibody Catlog	Present in Cohort 4
Channel 1	89Y	CD45	HI30	Biolegend	304002	Yes
Channel 2	115In	CD3	UCHT1	Biolegend	300443	Yes
Channel 3	141Pr	CD56	NCAM16.2	BD	559043	Yes
Channel 4	142Nd	TCR γ/δ	5A6.E9	PLT	100P001A	Yes
Channel 5	143Nd	Granzyme B Recombinant	QA16A02	Biolegend	372202	No
Channel 6	144Nd	CD14	M5E2	Biolegend	301862	Yes
Channel 7	145Nd	CD62L	DREG-56	Biolegend	304854	No
Channel 8	146Nd	CD197(CCR7)	G043H7	Biolegend	353256	Yes
Channel 9	147Sm	CD20	2H7	Biolegend	302302	No
Channel 10	148Nd	TIGIT(VSTM3)	A15153G	Biolegend	372702	No
Channel 11	149Sm	CD19	HIB19	Biolegend	302268	Yes
Channel 12	150Nd	IgD	IA6-2	Biolegend	348202	No
Channel 13	151Eu	CD161	HP-3G10	Biolegend	339902	No
Channel 14	152Sm	CD39	A1	Biolegend	328202	No
Channel 15	153Eu	CD27	O323	Biolegend	302802	No
Channel 16	154Sm	CD196(CCR6)	G034E3	Biolegend	353402	Yes
Channel 17	155Gd	CD185(CXCR5)	RF8B2	BD	552032	Yes
Channel 18	156Gd	CD127(IL-7Rα)	A019D5	Biolegend	351302	Yes
Channel 19	157Gd	CD183(CXCR3)	G025H7	Biolegend	353750	Yes
Channel 20	158Gd	CD11c	Bu15	Biolegend	337202	Yes
Channel 21	159Tb	CD33	WM53	Biolegend	303419	No
Channel 22	160Gd	CD28	CD28.2	Biolegend	302934	No
Channel 23	161Dy	CD45RA	HI100	Biolegend	304102	Yes
Channel 24	162Dy	CD69	FN50	Biolegend	310902	No
Channel 25	163Dy	T-bet	4B10	Biolegend	644802	No
Channel 26	164Dy	CD45RO	UCHL1	Biolegend	304202	Yes
Channel 27	165Ho	CD366(Tim-3)	F38-2E2	Biolegend	345010	Yes
Channel 28	166Er	CD95(Fas)	DX2	Biolegend	305656	Yes
Channel 29	167Er	CD25(IL-2Rα)	24212	RD	MAB1020	Yes
Channel 30	168Er	CD57	HNK-1	Biolegend	359602	Yes
Channel 31	169Tm	TCF1(TCF7)	7F11A10	Biolegend	655202	No
Channel 32	170Er	CD66b	G10F5	Biolegend	305102	Yes
Channel 33	171Yb	CD279(PD-1)	EH12.2H7	Biolegend	329926	Yes
Channel 34	172Yb	CD38	HIT2	Biolegend	303502	Yes
Channel 35	173Yb	CD194(CCR4)	L291H4	Biolegend	359402	Yes
Channel 36	174Yb	CD122(IL-2Rβ)	TU27	Biolegend	339004	No
Channel 37	175Lu	CD16	3G8	Biolegend	302057	Yes
Channel 38	176Yb	HLA-DR	L243	Biolegend	307648	No
Channel 39	197Au	CD4	RPA-T4	Biolegend	300541	Yes
Channel 40	198Pt	CD8a	RPA-T8	Biolegend	301074	Yes
Channel 41	209Bi	CD11b	M1/70	Biolegend	101202	No

Detailed iron channels information and antibodies information for CyTOF experiments.
